# Dysregulated phosphatidylinositol signaling promotes endoplasmic-reticulum-stress-mediated intestinal mucosal injury and inflammation in zebrafish

**DOI:** 10.1242/dmm.012864

**Published:** 2013-10-17

**Authors:** Prakash C. Thakur, Jon M. Davison, Carsten Stuckenholz, Lili Lu, Nathan Bahary

**Affiliations:** 1 Department of Medicine, Division of Hematology/Oncology, University of Pittsburgh Cancer Institute, Pittsburgh, PA 15232, USA.; 2 Department of Pathology, University of Pittsburgh School of Medicine, Pittsburgh, PA 15261, USA.; 3 Department of Microbiology and Molecular Genetics, University of Pittsburgh School of Medicine, Pittsburgh, PA 15219, USA.

**Keywords:** Cdipt, Phosphoinositides, IBD, UPR

## Abstract

Dysregulated phosphatidylinositol (PI) signaling has been implicated in human gastrointestinal (GI) malignancies and inflammatory states, underlining the need to study pathophysiological roles of PI in an *in vivo* genetic model. Here, we study the significance of PI in GI pathophysiology using the zebrafish mutant *cdipt^hi559^*, which lacks PI synthesis, and unravel a crucial role of PI in intestinal mucosal integrity and inflammation. The *cdipt^hi559^* mutants exhibit abnormal villous architecture and disorganized proliferation of intestinal epithelial cells (IECs), with pathologies reminiscent of inflammatory bowel disease (IBD), including apoptosis of goblet cells, abnormal mucosecretion, bacterial overgrowth and leukocyte infiltration. The mutant IECs exhibit vacuolation, microvillus atrophy and impaired proliferation. The *cdipt^hi559^* gene expression profile shows enrichment of acute phase response signaling, and the endoplasmic reticulum (ER) stress factors *hspa5* and *xbp1* are robustly activated in the mutant GI tissue. Temporal electron micrographic analyses reveal that PI-deficient IECs undergo sequential ER-Golgi disruption, mitochondrial depletion, macroautophagy and cell death, consistent with chronic ER-stress-mediated cytopathology. Furthermore, pharmacological induction of ER stress by inhibiting protein glycosylation or PI synthase inhibition in leukocyte-specific reporter lines replicates the *cdipt^hi559^* inflammatory phenotype, suggesting a fundamental role of PI metabolism and ER stress in mucosal inflammation. Antibiotics and anti-inflammatory drugs resolved the inflammation, but not the autophagic necroapoptosis of IECs, suggesting that bacterial overgrowth can exacerbate ER stress pathology, whereas persistent ER stress is sufficient to trigger inflammation. Interestingly, the intestinal phenotype was partially alleviated by chemical chaperones, suggesting their therapeutic potential. Using zebrafish genetic and pharmacological models, this study demonstrates a newly identified link between intracellular PI signaling and ER-stress-mediated mucosal inflammation. The zebrafish *cdipt* mutants provide a powerful tool for dissecting the fundamental mechanisms of ER-stress-mediated human GI diseases and a platform to develop molecularly targeted therapies.

## INTRODUCTION

Intestinal epithelial cells (IECs) play a major role in mucosal homeostasis, barrier function and immunity in addition to their digestive functions. Physiological stress to the IECs affects intestinal mucosal integrity, making the host susceptible to various gastrointestinal (GI) diseases. Epithelial disruption is a hallmark pathological feature of GI inflammatory disorders, particularly inflammatory bowel diseases (IBD) and necrotizing enterocolitis (NEC) (Xavier and Podolsky, 2007; Abraham and Cho, 2009; Henry and Moss, 2009).

Endoplasmic reticulum (ER) stress leading to epithelial dysfunction is believed to contribute to GI inflammation (Kaser and Blumberg, 2009; Hotamisligil, 2010; Kaser and Blumberg, 2010). ER stress results from perturbation of ER homeostasis through a multitude of factors and can trigger a conserved adaptive response, termed the unfolded protein response (UPR) or ER stress response (ERSR) (Ron and Walter, 2007). The UPR helps in protein folding capacity or ER-associated degradation (ERAD) of misfolded proteins to resolve the ER stress. However, chronic or unresolved ER stress, causing prolonged activation of UPR, can exacerbate the pathology of various human diseases (Lin et al., 2008). Recent studies on animal models of IBD have provided links between ERSR factors and GI inflammation. Mice lacking a crucial ERSR mediator, XBP-1, show cell-specific ER stress in the epithelium and develop spontaneous enteritis (Kaser et al., 2008). In humans, polymorphisms within the *XBP1* locus confer an increased risk for both Crohn’s disease (CD) and ulcerative colitis (UC) (Kaser et al., 2008). In murine models, ER-stress-mediated goblet cell (GC) depletion is implicated in the pathogenesis of UC (Heazlewood et al., 2008). Differential expression of the proximal ER stress sensor *HSPA5* is reported in human IBD tissues (Bogaert et al., 2011), and ER stress is hypothesized to activate pro-inflammatory signals through multiple mechanisms (Deng et al., 2004; Hu et al., 2006; Yamazaki et al., 2009). However, the precise molecular pathways leading to ER stress and the pathophysiological roles of various ERSR components in mucosal inflammation are largely unknown, necessitating the development of novel animal models to unravel these mechanisms. Furthermore, only a limited percentage of the estimated genetic heritability of IBD is explained by known genetic determinants identified by genome-wide association studies (Franke et al., 2010; Anderson et al., 2011), indicating the importance of finding new animal models to delineate specific genes and pathways that might contribute to IBD pathogenesis.

The zebrafish, *Danio rerio*, has been an effective tool in deciphering mechanisms of human GI diseases, due to the similarity in basic GI tissue structure, function and gene expression profiles (Stuckenholz et al., 2004; Stuckenholz et al., 2009). The zebrafish intestine is fully developed and becomes functional by 5 days post-fertilization (dpf), displaying a villous architecture with easily identifiable enterocytes, enteroendocrine cells, and the mucin-secreting GCs (Wallace and Pack, 2003; Ng et al., 2005; Stuckenholz et al., 2004). Zebrafish IECs secrete defensins and other antimicrobial peptides, and the IBD susceptibility genes *nod1* and *nod2* have been shown to maintain conserved antimicrobial roles in the zebrafish intestine (Oehlers et al., 2011a; Oehlers et al., 2011b). Chemical enterocolitis models in the zebrafish have shown that features of IBD seen in murine models can be rapidly recapitulated in larval zebrafish, emphasizing their utility for the study of IBD pathogenesis (Fleming et al., 2010). In addition, larval zebrafish models are being utilized to analyze interactions between the commensal microbiota and host innate immunity, providing insights into the role of bacteria and inflammation in human IBD (Kanther et al., 2011; Roeselers et al., 2011).

**TRANSLATIONAL IMPACT**

**Clinical issue**

Intestinal epithelial disruption and inflammation is a hallmark feature of several chronic gastrointestinal diseases, including inflammatory bowel disease (IBD) and cancers of the gastrointestinal tract. IBD is a debilitating chronic disorder, with a peak incidence in early adult life, that often requires lifetime prescriptions of drugs that cause significant side effects. Although IBD is believed to result from an inappropriate inflammatory response to commensal microbes in a genetically susceptible host, we only have limited insights into its pathogenesis, underlining the importance of finding novel genes and pathways that might contribute to the inflammatory process. Notably, genes that affect the cellular stress response pathway have recently been implicated in IBD pathogenesis. Zebrafish provide an attractive tool for unraveling the underlying mechanisms in gastrointestinal disease, given the similarity with the mammalian system in terms of the basic architecture of the digestive system, cell types and function. The model also allows *in vivo* imaging and high-throughput drug screening.

**Results**

To explore the underlying mechanisms driving cellular stress and inflammation, the authors used a zebrafish genetic model linking phosphatidylinositol (PI) signaling to these processes, as PI signaling is known to be associated with a number of gastrointestinal diseases and malignancies. The authors used *cdipt^hi559^* zebrafish, which are deficient in *de novo* PI synthesis, to elucidate the importance of PI signaling in gastrointestinal physiology. Mutant zebrafish demonstrated persistent ER stress and disrupted intestinal architecture, epithelial restitution and homeostasis. The unresolved ER stress sequentially leads to reduced mucosecretion, goblet cell apoptosis, autophagy, bacterial overgrowth and myeloid inflammation in the mucosa, resembling IBD pathologies. The authors show that pharmacological induction of ER stress is sufficient to elicit similar inflammatory phenotypes. Interestingly, suppression of inflammation by anti-inflammatory drugs failed to resolve the ER stress pathologies, whereas ER stress alleviation by chemical chaperones resolved the mutant phenotype.

**Implications and future directions**

Using a whole organism *in vivo* approach, this study unravels novel mechanistic insights into the pathophysiology of gastrointestinal diseases. The data described provide the first evidence to link a deficiency in PI synthesis with ER-stress-mediated intestinal mucosal injury and inflammation. The ER homeostasis and inflammatory pathways appear to be conserved between zebrafish and humans, suggesting that modulation of PI signaling and ER stress components might alleviate gastrointestinal inflammation. This work thereby provides new avenues for therapeutic strategies to treat IBD and associated diseases. The zebrafish genetic and pharmacological model presented here is amenable to treatment with commonly tested anti-inflammatory drugs and chemical chaperones, indicating that it can be used as a preclinical platform to develop molecularly targeted therapies for gut-related inflammatory diseases and cancer.

Phosphatidylinositol (PI) signaling has been linked to a variety of human diseases and cancer. PI is a crucial phospholipid synthesized in the ER and in highly dynamic ER-derived compartments. PI is rapidly metabolized and its levels are tightly controlled in the cell to exert its spatiotemporal intracellular signaling functions (Balla et al., 2009; Kim et al., 2011). Phosphorylated PIs (PIPs) are believed to be the regulators of vesicular transport and secretory pathways. We have previously shown by transcriptome profiling that inositol metabolism and phosphoinositide 3-kinase (PI3-K) pathways are enriched during zebrafish GI development and that inhibition of PI3-K signaling results in GI developmental defects (Stuckenholz et al., 2009). To further define the pathophysiological significance of intracellular PI, we identified and characterized the zebrafish insertional mutant *cdipt^hi559^* (*hi559*), which is defective in PI synthesis. Cdipt (CDP-diacylglycerol–inositol 3-phosphatidyltransferase) is a highly conserved enzyme with its active site on the cytoplasmic face of the ER and is responsible for synthesis of intracellular PI from myo-inositol and CDP-diacylglycerol. PI synthesis has been suggested to occur in a dynamic domain of the ER positioned at the leading edge of the ER tubules (English and Voeltz, 2013). Our earlier studies characterizing the *hi559* mutation showed that the lack of *de novo* PI synthesis leads to ER stress and hepatic steatosis (Thakur et al., 2011). Despite the ubiquitous need for PI, the *hi559* mutation does not cause a broad, general developmental defect. The enrichment of Cdipt expression in the larval GI tissues accounts for the strong GI defects in *hi559* larvae due to functional loss of Cdipt and consequent deficiency of *de novo* PI synthesis, in spite of maternally deposited PI in the yolk. Given the transient roles and dynamics of PI metabolism, it is conceivable that Cdipt-controlled *de novo* PI synthesis is crucial for intracellular availability of PIPs such as phosphatidylinositol 4-phosphate [PI(4)P] and phosphatidylinositol (4,5)-bisphosphate [PI(4,5)P2] to exert their secretory functions.

The *cdipt* mutant zebrafish develop consistent GI defects during late larval stages after tissue differentiation, exhibiting a complex pathology in the intestine: abnormal IEC proliferation and apoptosis, villous atrophy, GC depletion, bacterial overgrowth and inflammation, all of which are hallmarks of human IBD. The *hi559* IECs show disruption of ER architecture followed by mitochondrial defects and increased autophagy and cell death, consistent with ER-stress-induced cytopathology. Pharmacological induction of ER stress in wild-type larvae results in similar inflammatory pathologies, suggesting a contributory role of aberrant PI synthesis in ER-stress-mediated GI inflammation. In addition, akin to IBD treatment strategies, the mutant phenotype is partially ameliorated by antibiotic and anti-inflammatory drugs. This highlights the utility of this system as a tool for studying the pathogenesis of ER stress and mucosal inflammation. These studies facilitate novel insights into the mechanistic relationships between intracellular PI signaling, ER stress and GI pathophysiology in a whole-organism *in vivo* setting.

## RESULTS

### Loss of phosphatidylinositol synthase causes defects in intestinal architecture

**Fig. 1.**
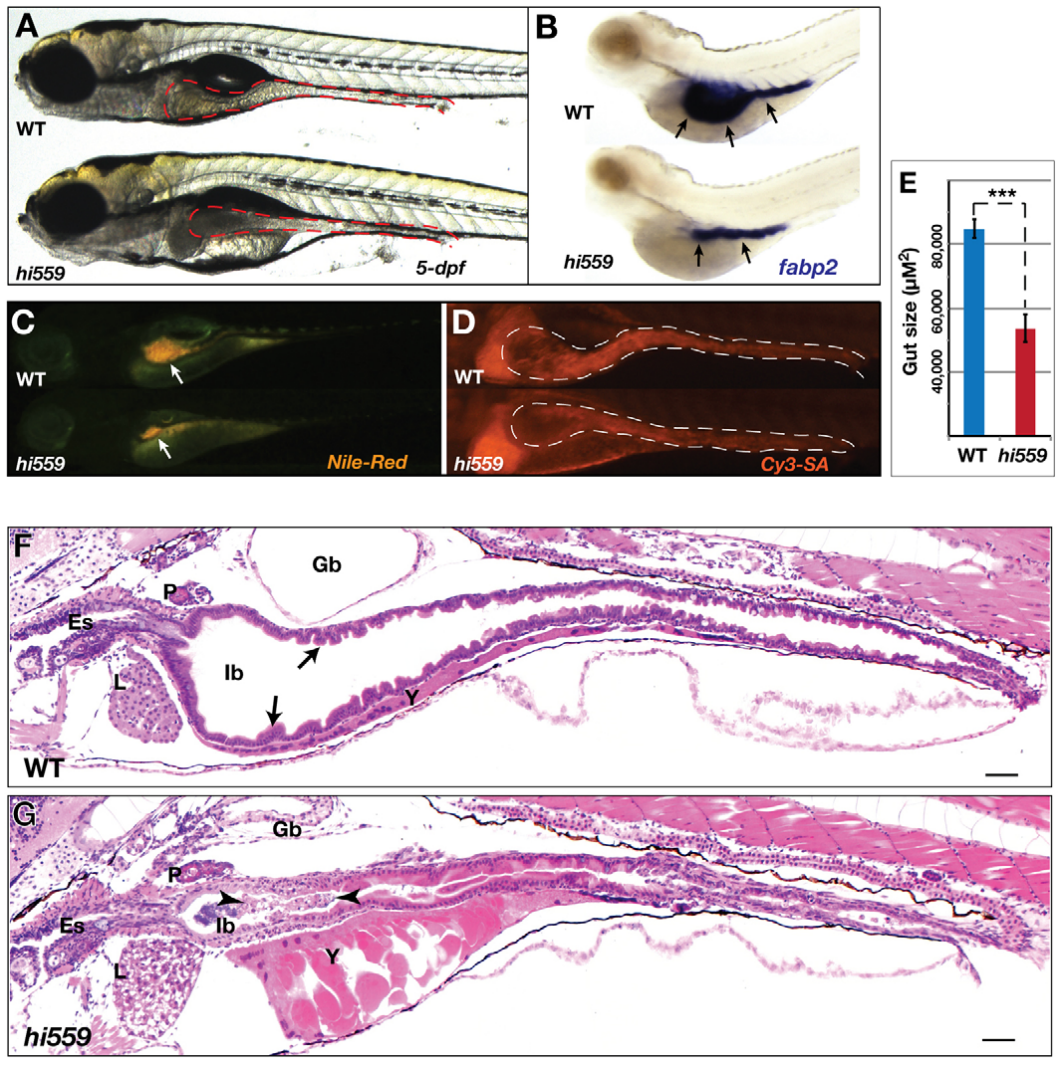
**Morphological defects of *hi559* GI tract.** (A) Intestinal morphology at 5 dpf (brightfield; red outline). (B) ISH with intestinal marker *fabp2* (arrows) at 5 dpf. (C) Nile Red staining shows *hi559* intestinal luminal atrophy (arrows). (D) Cy3-SA staining shows reduced epithelial structure in *hi559* intestine (white outline). (E) Bar chart showing reduced gut size in *hi559Tg(gut:gfp)* mutant larvae show smaller intestine (*n*=7, ***P<0.001). (F,G) H&E-stained sagittal sections of 5-dpf wild-type (F) and *hi559* larvae (G). The *hi559* intestinal epithelium is thinner, loses villous architecture with cellular aggregates in a smaller lumen (villi, arrows; cells and debris, arrowheads). In each panel, wild type (WT) is shown at the top and the *hi559* mutant below. Es, esophagus; Gb, gas-bladder; Ib, intestinal bulb; P, pancreas; L, liver; Y, yolk; cm, cell membrane. Scale bars: 20 μm.

The *hi559* homozygous mutant lacks Cdipt expression due to a retroviral insertion within the *cdipt* gene. Larvae develop normally until 5 dpf, when they begin to exhibit hepatic defects, including hepatomegaly and steatosis (Thakur et al., 2011). Another striking feature of the mutant is a smaller intestine by 5 dpf (Fig. 1A). Analyses of *hi559* larvae expressing green fluorescent protein (GFP) in the gut [*hi559Tg(gut:gfp)*] confirmed that the *hi559* mutation is fully penetrant, consistently presenting with a significantly smaller intestine at 5 dpf (*P*<0.001; Fig. 1E). Homozygous mutant larvae die at larval stages between 6.5 and 7 dpf. To prove that loss of Cdipt and its PI synthesis function underlies the mutant GI phenotype, we showed that knockdown of Cdipt by morpholino injection into wild-type embryos or chemical inhibition of PI synthesis in wild-type larvae by δ-hexachlorocyclohexane (δ-HCH) replicates the *hi559* phenotype (supplementary material Fig. S1E-G) (Thakur et al., 2011). In addition, larvae with a weaker Cdipt mutant allele, *cdipt^lop^* (*lop*) (Murphy et al., 2011), which carries a point mutation in the phosphatidylinositol synthase (PIS) domain, also replicated the *hi559* GI phenotype (supplementary material Fig. S1C), but exhibited a milder, delayed phenotype. They developed normally until 7 dpf, subsequently exhibiting similar gross and histological intestinal abnormalities as seen in *hi559* larvae and dying at about 10 dpf. Both *hi559* and *lop* failed to rescue each other in a complementation assay (supplementary material Fig. S1D), supporting the conclusion that *lop* is a hypomorphic allele of *hi559*. In homozygous *hi559* and *lop* mutants, Cdipt function is eliminated, resulting in abrogation of *de novo* PI synthesis (Murphy et al., 2011; Thakur et al., 2011). The similarity of the intestinal abnormalities in both *lop* and *hi559* larvae suggest that the mutant phenotype is not primarily an early developmental defect, but reflects a requirement for *de novo* PI in intestinal function at later larval stages. Because *hi559* mutants offer the advantage of an earlier and consistently stronger GI phenotype, we used them to elucidate the role of PI in GI tissues.

The hypomorphic nature of *hi559* intestine is evident from whole-mount staining and histology (Fig. 1A-G; supplementary material Fig. S1). Incorporation of Nile Red was diminished in the mutants, indicating decreased luminal volume (Fig. 1C; supplementary material Fig. S1). The intestinal epithelial structure was reduced in size, as demonstrated by Cy3-streptavadin (Cy3-SA) immunostaining (Fig. 1D), suggesting that the hypomorphic intestinal manifests itself in both smaller lumen and thinner epithelium. To analyze developmental abnormalities in the intestine, we characterized the *hi559* larvae by whole-mount *in situ* hybridization (ISH) using RNA probes against the intestine-specific markers *fabp2*, *vil1* and *anxa2b* (Fig. 1B; supplementary material Fig. S1A). The observed decrease in marker gene expression suggests loss of structural and functional components of the *hi559* intestine by 5 dpf. There was no difference in expression of intestinal markers or Nile Red staining until 4 dpf in *hi559* compared with wild type (data not shown), suggesting no gross physical defects during early intestinal development. Intestinal expression of *cdipt* in wild-type larvae (Thakur et al., 2011) and the intestinal defects of *hi559* and *lop* larvae (Fig. 1; supplementary material Fig. S1) implicate an important role of PIS in intestinal integrity and function.

### The *hi559* intestine exhibits abnormal villous architecture and mucosal cells

**Fig. 2.**
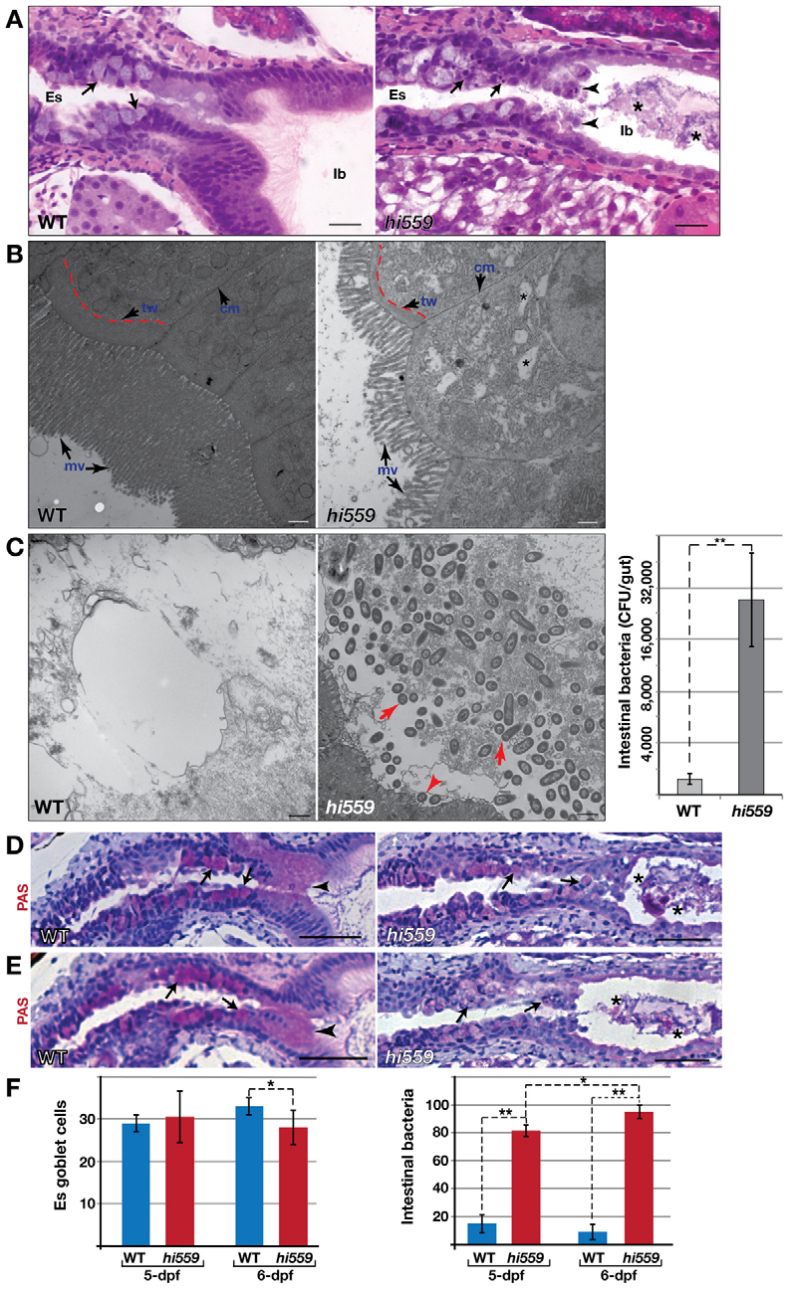
**Disrupted epithelial architecture, abnormal IEC and increased luminal bacteria in *hi559* intestine.** (A) In *hi559* anterior GI tract, the IECs appear less columnar, a few IECs detach from the mucosa (arrowheads) and the esophageal GCs (arrows) appear disorganized with nuclear pyknosis. Asterisks indicate luminal bacterial plaques. (B) TEM comparison of intestinal epithelium of 6-dpf wild type (WT, left) and *hi559* (right). Wild-type intestine shows columnar IECs, thick terminal web (tw; red line) and long microvilli (mv; arrows). *hi559* IECs have thinner terminal webs, shorter microvilli and increased cytoplasmic vacuoles (asterisks). (C) TEM showing dense bacterial colonies in *hi559* intestinal lumen (red arrows), but not in wild type. Bar chart (right) shows significant increase in intestinal bacterial density in *hi559* (*n*=5). (D,E) The mucin-rich esophageal GCs (arrows) at 5 dpf (D) and 6 dpf (E) are shown by PAS staining (pink). The secreted mucinous layer (arrowhead) on the epithelial border seen in the wild type is diminished in *hi559* with frequent detachment of GCs. Asterisks indicate luminal bacterial plaques. (F) Bar charts showing PAS-positive GC numbers in 5- and 6-dpf wild-type and *hi559* esophagus (left; *n*=7) and the percentage of larvae with intra-luminal bacterial overgrowth at 5 and 6 dpf (right; *n*≥21). Es, esophagus; Ib, intestinal bulb. *P<0.05, **P<0.01. Scale bars: 20 μM (A,D,E); 500 nm (B,C).

Histological examination showed that the villous and luminal architecture was disrupted in the *hi559* intestine (Fig. 1F,G). In wild-type larvae, columnar IECs are well polarized, forming a continuous epithelial monolayer with villi. In contrast, *hi559* IECs were disorganized, less columnar with incomplete cytoplasmic maturity, and sporadically detached from the epithelium into the lumen (Fig. 1F,G and Fig. 2A). The intermittently detached IECs had nuclear condensation, suggesting apoptosis (supplementary material Fig. S2A). TEM analysis of the 5.5-dpf intestinal mucosa demonstrated that the wild-type IECs exhibit a highly elaborate apical brush border with microvilli projecting into the lumen, whereas the *hi559* IECs had enlarged cytoplasmic vesicles, abnormal brush border, reduced terminal web and microvillus atrophy (Fig. 2B). The *hi559* intestinal lumen was consistently filled with basophilic plaques (Fig. 2A), which TEM and colony formation assays confirmed to be largely due to increased bacterial colonization (*P*=0.0027; Fig. 2C).

Mucin-secreting GCs in the esophageal and mid-intestinal regions are typically evident in zebrafish larvae by 5 dpf. In *hi559*, these cells appeared abnormal with pyknotic or fragmented nuclei, suggesting apoptosis (Fig. 2A). We used PAS staining and TEM to analyze these GCs. In wild-type intestine, a thick secreted mucinous layer was consistently seen covering the apical border of the epithelium, which was diminished in the *hi559* GI tract, suggesting alteration of GC physiology and their secretory function (Fig. 2D,E). Ultrastructurally, the 5-dpf wild-type GCs showed mature theca containing large mucinous vacuoles. In *hi559*, these appeared immature and degenerated (supplementary material Fig. S2B). Interestingly, there was no difference in numbers of GCs at 5 dpf (*P*=0.667), supporting normal IEC differentiation. However, the population of esophageal GCs declined by 6 dpf (*P*=0.0456; Fig. 2D–F) due to apoptosis and detachment. Mid-intestinal GCs and mucus secretion were similarly depleted in *hi559* (*P*=0.0489; supplementary material Fig. S2C–E). Concomitant with GC depletion, nearly all *hi559* larvae (>90%) showed histological features of bacterial overgrowth in the intestine by 6 dpf (*P*=0.002; Fig. 2F).

### Abnormal proliferation and apoptosis of the *hi559* IECs

**Fig. 3.**
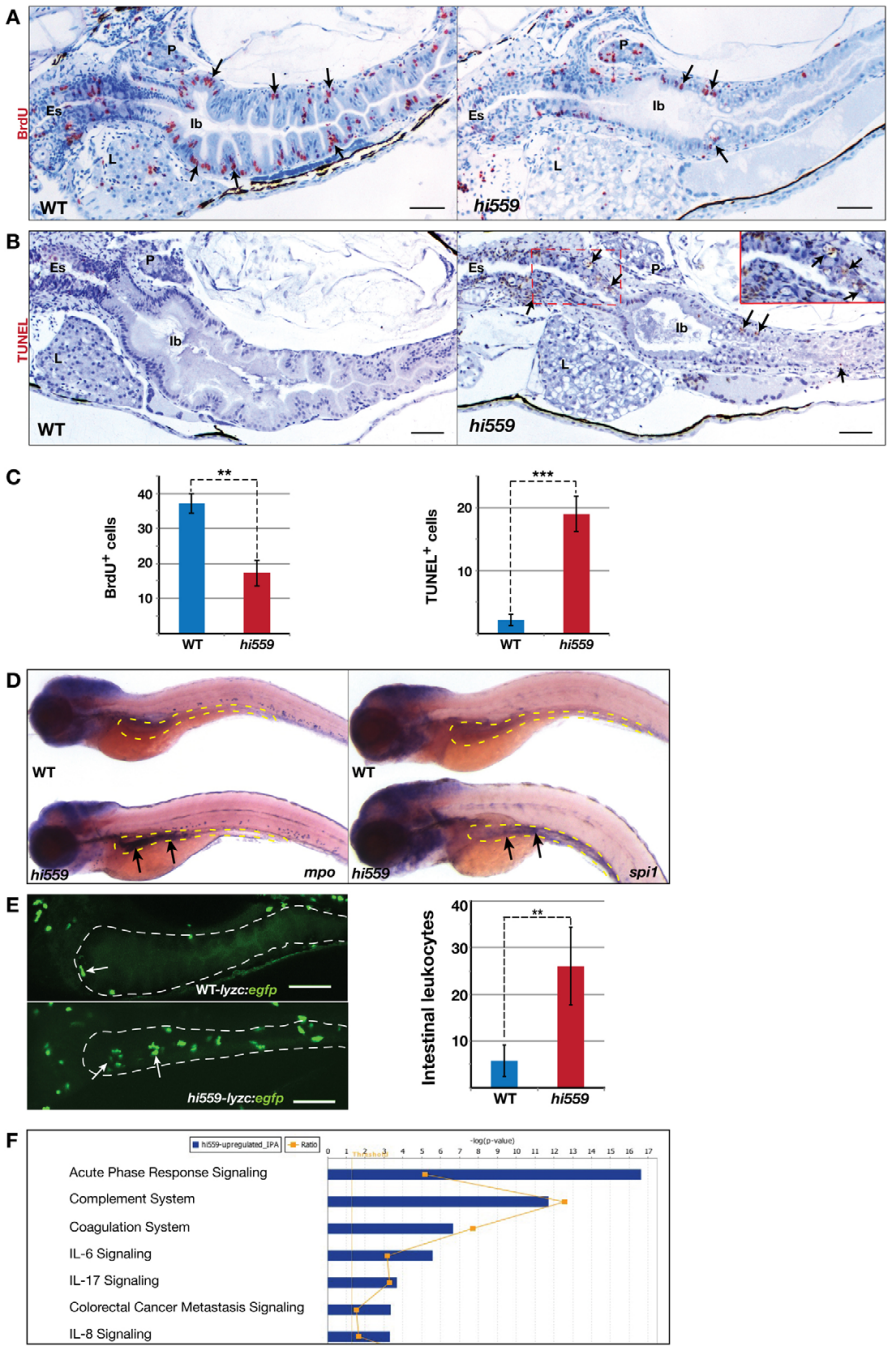
**Abnormal cell proliferation, apoptosis and inflammation in *hi559* intestine.** (A) BrdU staining (red) shows decreased proportion of proliferating cells (arrows) in the *hi559* intestine compared with wild type (WT). (B) TUNEL staining (brown) shows several apoptotic cells in *hi559* GI tract (red box indicates esophageal GC region magnified in inset). (C) Bar charts showing the proportion of BrdU-positive cells at 5 dpf (left), and TUNEL-positive cells at 6 dpf (right; *n*=8). (D) ISH showing increased expression (arrows) of neutrophil marker *mpo* (left) and macrophage marker *spi1* (right) in *hi559* intestines (yellow outline) at 6 dpf. (E) Confocal projections of 6-dpf *Tg(lyzc:egfp)* and *hi559Tg(lyzc:egfp)* larval intestines (white outline), showing leukocyte aggregation (arrows). Bar chart shows the number of leukocytes in wild-type and *hi559* intestines at 6 dpf (*n*=12). (F) IPA analysis of microarray profile showing most significantly upregulated pathways in *hi559* larvae (*n*=3, *P*≤0.01). Es, esophagus; Ib, intestinal bulb; P, pancreas; L, liver. **P<0.01, ***P<0.001. Scale bars: 20 μm.

We studied the fate of the IECs by analyzing their cell-cycle status by BrdU incorporation and TUNEL assays. In the wild-type intestine, BrdU-positive cells occur frequently, typically at the base of the epithelial villi; TUNEL-positive cells are rare (Fig. 3A,B). Although BrdU labeling and TUNEL assays did not reveal abnormal cell proliferation or apoptosis in the *hi559* GI tract prior to 5 dpf, IEC proliferation was significantly reduced and disorganized at 5 dpf (*P*=0.006; Fig. 3A,C) and the frequency of apoptotic cells increased as the *hi559* intestinal pathology worsened by 6 dpf (*P*=0.0003; Fig. 3B,C), resulting in focal ulceration of the intestinal epithelium. TUNEL assays confirmed that the GCs with pyknotic nuclei observed in the *hi559* GI tract were predominantly undergoing apoptosis, which might account for their depletion (Fig. 3B,C). In the disorganized epithelial region, IECs are often largely vacuolated (supplementary material Fig. S3A). These vacuoles did not show PAS or Oil-Red-O staining, suggesting that they were neither mucinous nor steatotic (data not shown). Taking the results together, we conclude that PI deficiency impedes proliferation and induces apoptosis of IECs, causing villous atrophy and intestinal hypoplasia.

### PI deficiency causes mucosal pathology and inflammation with IBD-like features

During larval development, the *hi559* intestine exhibited increasing bacterial overgrowth coinciding with depletion of the GCs (Fig. 2F). Because loss of mucinous secretions and aberrant bacterial growth in the gut can cause spontaneous inflammation, as seen in IBD, we assayed intestinal inflammation in *hi559* larvae. Onset of an inflammatory response was evident at 5.5-6 dpf, as ISH demonstrated infiltration of the epithelium with *mpo*-positive neutrophils and *spi1*-positive macrophages (Fig. 3D). Necro-inflammatory injury was evident histologically by 6 dpf (supplementary material Fig. S3A). To quantify leukocyte infiltration, we utilized the leukocyte-specific reporter line *Tg(lyzc:egfp)*. Analysis of *hi559 Tg(lyzc:egfp)* mutant larvae revealed significantly higher leukocyte aggregation in the 6-dpf intestine compared with wild-type *Tg(lyzc:egfp)* (*P*=0.008; Fig. 3E). Interestingly, pharmacological inhibition of PI synthesis by the chemical inhibitor δ-HCH in wild-type larvae replicated the increased intestinal leukocyte aggregation seen in *hi559* (*P*=0.031; supplementary material Fig. S3B), further substantiating the hypothesis that deficient PI synthesis leads to mucosal inflammation.

Pathway analysis of *hi559* gene expression identified acute phase response (APR) signaling as the most significantly upregulated canonical pathway, suggesting activated transcription of pro-inflammatory factors (*P*=0.002; Fig. 3F). In addition to the complement cascade pro-inflammatory factors, interleukin (IL-6, IL-8 and IL-17) signaling and NF-κB signaling were among the most significantly dysregulated gene sets, suggesting that pro-inflammatory activity might be mediated via these pathways (*P*≤0.01; Fig. 3F and supplementary material Fig. S4A,B).

### Zebrafish deficient in *cdipt* exhibit unresolved ER stress and macroautophagy in IECs

**Fig. 4.**
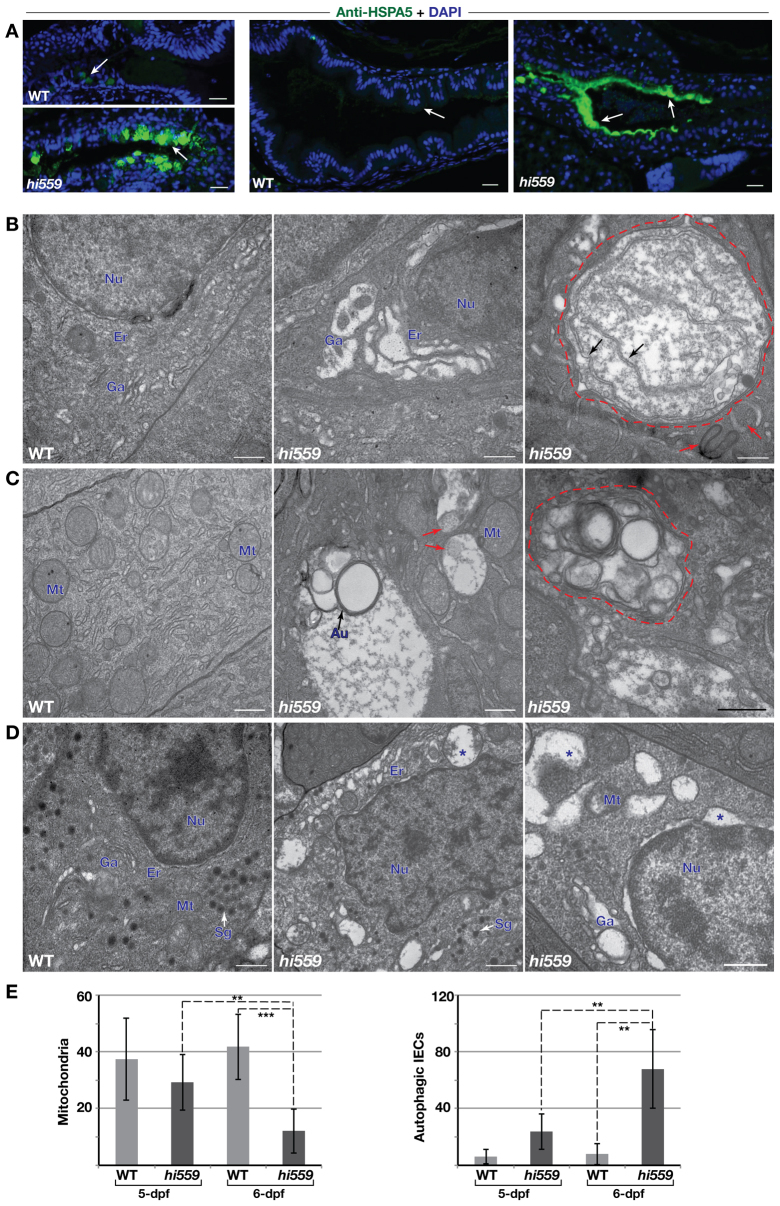
**ER stress and ultrastructural pathology of IECs.** (A) Anti-HSPA5 immunofluorescence assay (green) shows robust enrichment of Hspa5 protein in the GCs (arrows, left panel) and the IECs along the epithelial lining (arrows, right panel) of *hi559* intestine compared with wild type (WT). (B-D) TEM comparison of wild type (left panels) and *hi559* IECs (middle and right panels). (B) ER-Golgi compartments are grossly expanded in 5-dpf *hi559* IECs. Large double-membranous autophagic vacoules (red outline) and pre-autophagosome structures (red arrows), containing ER fragments (black arrows) are apparent in 5.5-dpf *hi559* IECs (right panel). (C) Wild-type IECs have abundant mitochondria, whereas *hi559* IECs have depleted, abnormal mitochondria and increased mitophagy at 6 dpf (red arrows). Multi-lamellar autophagic bodies (red outline), engulfing organelles, occur frequently in *hi559* IECs at 6 dpf. (D) Secretory granule-rich enteroendocrine cells show ER luminal swelling (asterisks) and autophagic vesicles in *hi559*. Nu, nucleus; Er: endoplasmic reticulum; Ga, Golgi apparatus; Au, autophagosome, Sg, secretory granules. Mt, mitochondria. (E) Bar charts of mitochondrial (left) and autophagosome (right) counts in IECs (*n*=7); **P<0.01, ***P<0.001. Scale bars: 20 μm (A); 500 nm (B-D).

Because our microarray-based pathway analyses revealed enrichment of ERSR gene sets in *hi559* larvae (Thakur et al., 2011), we further investigated the nature of ER stress pathology in the GI tract. Unresolved ER stress is typically marked by persistent UPR induction and subsequent disruption of ER architecture and function. HSPA5 (also known as GRP78) is a heat shock protein that chaperones proteins in the ER lumen and is upregulated in response to ER stress (Marciniak and Ron, 2006), and *XBP1* mRNA splicing is a key marker of ER stress and UPR activation (Calfon et al., 2002). The expression of proximal ERSR sensors *hspa5* and *xbp1* was robustly elevated in the *hi559* GI tract as seen by ISH (supplementary material Fig. S5A,B) (Thakur et al., 2011). In addition, splicing of *xbp1* was evident in the micro-dissected GI tissue of *hi559* larvae by RT-PCR (supplementary material Fig. S5C). The elevation of both unspliced and spliced transcripts of *xbp1* indicated that there was ongoing ER stress in the *hi559* GI tissues. To further clarify the ER stress within tissues, we performed immunohistochemistry to detect active Hspa5 protein. Robust enrichment of Hspa5 protein was seen within *hi559* GI tissues, specifically in the mucin secreting GCs and subsequently in the IECs of the intestinal mucosa (Fig. 4A).

Because these ER stress UPR factors are associated with molecular pathogenesis of IBD (Kaser et al., 2008; Bogaert et al., 2011), we wanted to further analyze the temporal ultrastructural defects within *hi559* GI tissues to dissect the sequence of ER-stress-mediated pathology at cellular level. We performed extensive TEM analysis of the intestinal mucosa at different stages of the phenotype (Fig. 4B,D). At 5 dpf, the most striking defect in *hi559* IECs was a disruption of the ER-Golgi architecture, without any overt changes in other cellular components (Fig. 4B). Large double-membrane macroautophagic bodies (autophagosomes) causing focal cytoplasmic necrosis were evident by 5.5 dpf (Fig. 4B). At 6 dpf, the IECs showed extensive mitophagy, depletion of mitochondria, and large multilamellar autophagosomes containing cytoplasmic organelles (Fig. 4C). The lumens of the distended ER-Golgi compartments in the *hi559* IECs were often filled with aggregates of variable electron density, suggesting protein accumulation. Significantly increased autophagy and loss of mitochondria could account for the large cytoplasmic vesicles of 6-dpf IECs (*P*≤0.003; Fig. 4E). ER-stress-associated cytopathology and autophagic vesicles were also evident in the secretory enteroendocrine cells (Fig. 4D). ER expansion and autophagy occurred in both the pancreatic endocrine cells and the zymogen-rich acinar cells by 6 dpf, suggesting that the ER-stress-induced pathology is subsequently propagated in the majority of the secretory cells of the digestive system (supplementary material Fig. S5D,E). Disrupted ER architecture, grossly expanded ER lumens and vacuolization, consequent mitochondrial damage, and autophagy are consistent with ER-stress-induced cytopathology. These results demonstrate that unresolved ER stress in the highly secretory GI cells is the major etiology of the *hi559* phenotype, implying that the lack of *de novo* PI impedes secretory function, leading to pathological ER-stress-induced GI defects in *cdipt* mutants.

### ER stress is causal and sufficient to the induction of GI inflammatory pathology

**Fig. 5.**
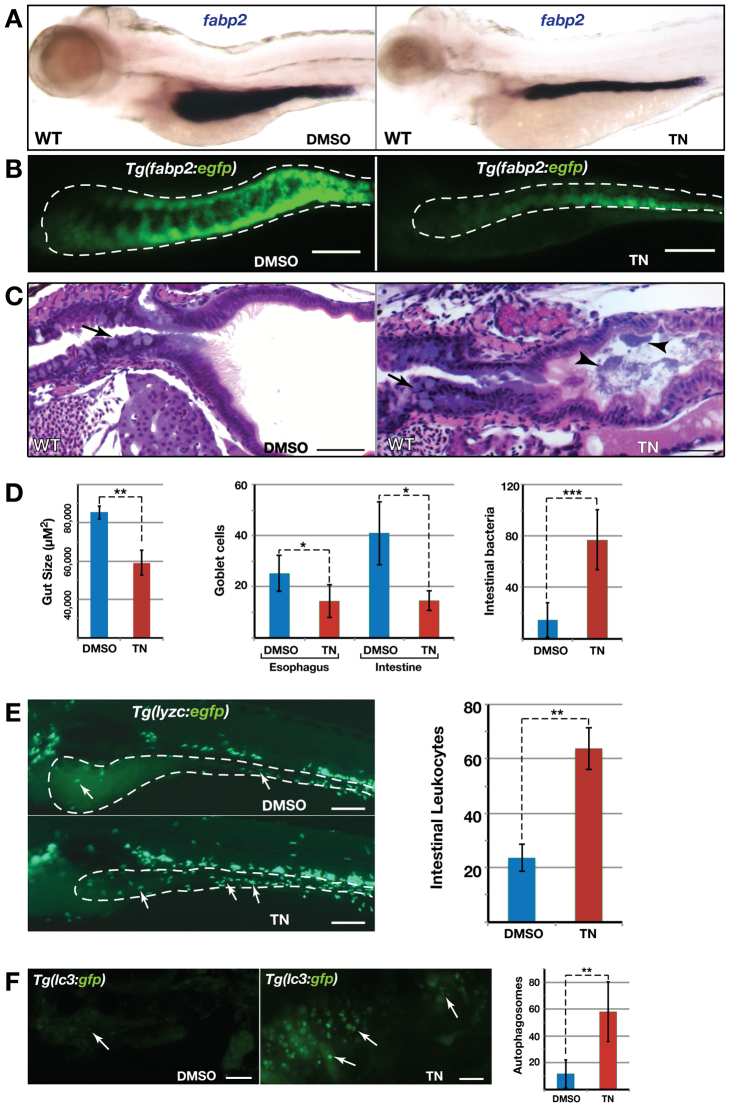
**Intestinal pathologies of tunicamycin-treated wild-type larvae.** (A) ISH with *fabp2* shows smaller intestine in tunicamycin (TN)-treated larvae. (B) Confocal projections of *Tg(fabp2:egfp)* intestine (white outline) showing disrupted intestinal architecture of tunicamycin-treated larvae. (C) H&E-stained sections shows abnormal GCs (arrows) and increased luminal bacteria (arrowheads) in tunicamycin-treated larvae. (D) Bar charts showing reduced gut size, GC depletion and increased intestinal bacteria in tunicamycin-treated larvae (*n*≥10). (E) Confocal projection of *Tg(lyzc:egfp)* larval intestine shows increased macrophage aggregation (arrows) in tunicamycin-treated larvae (*n*=15). (F) Confocal projection of *Tg(lc3:gfp)* intestine shows increased autophagosomes (arrows) in tunicamycin-treated larvae (*n*=7). *P<0.05, **P<0.01, ***P<0.001. Scale bars: 100 μm (B,E); 20 μm (C); 5 μm (F).

Our analysis of *hi559* mutants has not yet addressed the sequence of events leading to the overt phenotype: is the ER stress a direct consequence of functional loss of Cdipt, or induced by another unrecognized process such as inflammation? To help distinguish between these possibilities, we tested whether tunicamycin, a compound known to induce ER stress by inhibition of N-glycosylation, can cause GI inflammation. Chronic treatment of wild-type zebrafish with 1 μM tunicamycin from 3.5 dpf through to 6 dpf resulted in a smaller intestine (*P*=0.002) and defects in intestinal architecture (Fig. 5A–C and supplementary material Fig. S6A). Interestingly, tunicamycin-treated larvae exhibited increased bacterial growth (*P*=0.0006), GC depletion (*P*≤0.02), and increased intestinal macrophage and neutrophil infiltration (*P*≤0.006; Fig. 5C–E and supplementary material Fig. S6B,D). Necro-inflammatory lesions containing large numbers of vacuolated or autophagic IECs in tunicamycin-treated *Tg(lc3-gfp)* larval intestines were clearly evident (*P*=0.005; Fig. 5F and supplementary material Fig. S6C). These results clearly suggest that chronic ER stress is sufficient to trigger necro-inflammatory injuries in the zebrafish intestine, leading to *hi559*-like GI pathology.

### Anti-inflammatory drugs and chemical chaperones ameliorate ER-stress-induced GI inflammation

Recruited macrophages and neutrophils are potent sources of cytokines and tissue destructive enzymes, contributing to necro-inflammatory injury by loss of IEC integrity. Anti-inflammatory agents, such as 5-aminosalicylic acid (5-ASA) and prednisolone, are thus widely used therapies for alleviating human inflammatory disorders, and remain an important option for treating patients presenting with moderate to severe IBD. In recent years, co-administration of antibiotics or probiotics with anti-inflammatory drugs is also being prescribed as an effective regimen for IBD treatment (Perencevich and Burakoff, 2006). To illustrate the similarity of *cdipt* mutants to human inflammatory states and as an *in vivo* system in which to assay potential suppressors of the inflammatory pathology, we assessed the response of *hi559* mutants to antibiotics and anti-inflammatory drugs.

**Fig. 6.**
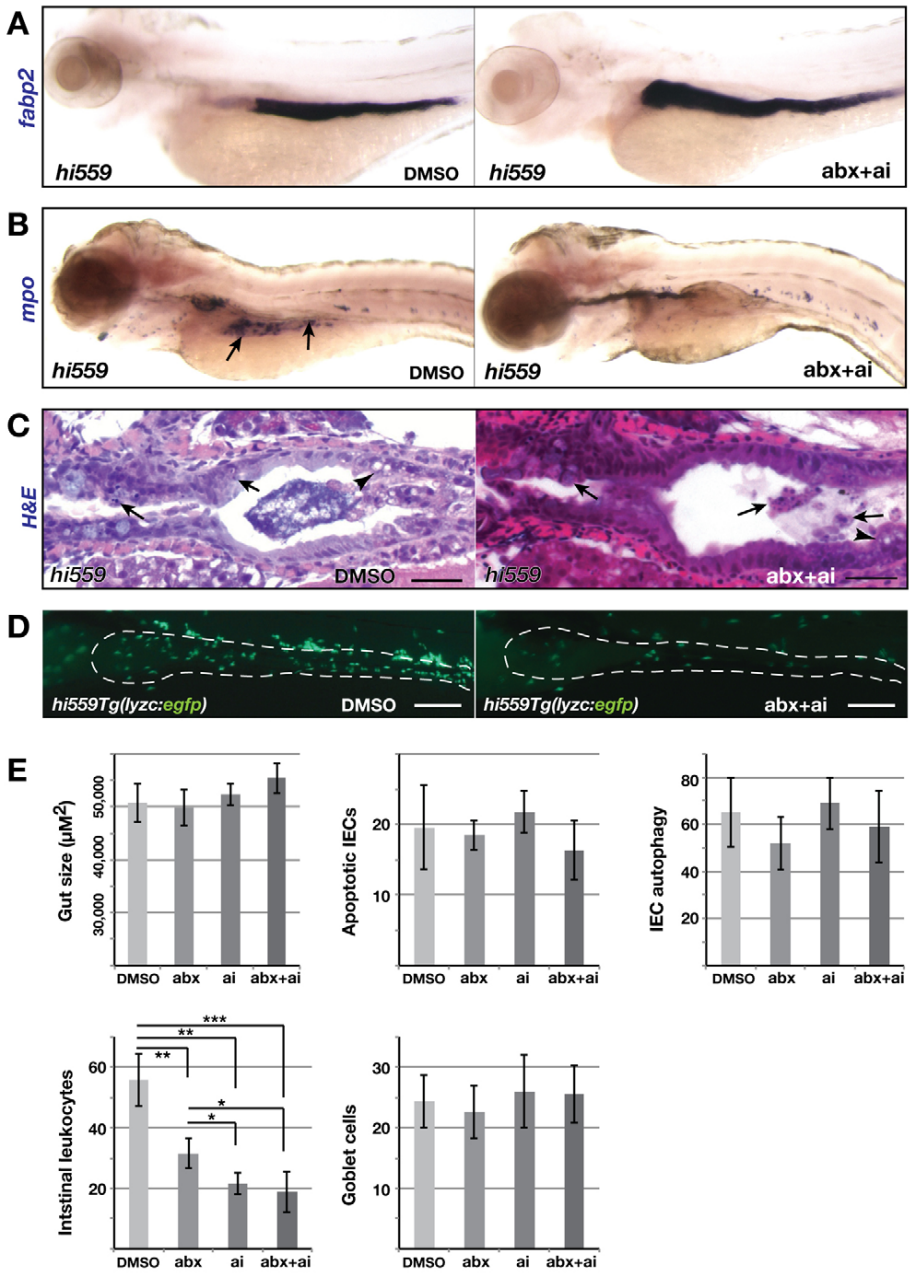
**Suppression of inflammation by antibiotics and anti-inflammatory drugs.** Larvae were treated with antibiotics (abx) and anti-inflammatory (ai) drugs from 3.5 to 6 dpf. (A) ISH with *fabp2* shows improvement of intestinal architecture in *hi559* larvae treated with combined antibiotics and anti-inflammatory drugs. (B) ISH with *mpo* shows reduction of intestinal neutrophil infiltration (arrows) in drug-treated *hi559* larvae. (C) H&E-staining shows reduction of luminal bacteria and inflammation in drug-treated *hi559* larvae. IECs vacuolation (arrowheads), apoptosis and shedding (arrows) are seen in both DMSO and drug-treated larvae. (D) Fluorescent micrograph of drug-treated *hi559Tg(lyzc:egfp)* mutant larvae shows reduced intestinal leukocyte infiltration. (E) Bar charts show gut size, percentages of apoptotic IECs, autophagic IECs, intestinal leukocyte counts and esophageal GCs in DMSO- or drug-treated *hi559* larvae (*n*≥12). *P<0.05, **P<0.01, ***P<0.001. Scale bars: 20 μm (C); 100 μm (D).

Treatment with antibiotics or anti-inflammatory drugs alone failed to rescue the *hi559* intestinal phenotype and did not increase gut size (*P*=0.72 and 0.39, respectively), although leukocyte infiltration was effectively reduced (*P*≤0.01; Fig. 6E; supplementary material Fig. S7A–C). Co-administration of anti-inflammatory drugs 5-ASA and prednisolone together with antibiotics from 3.5 to 6 dpf resulted in marginal alleviation of *hi559* intestinal size, as seen by ISH with *fabp2*, showing a minor increase in gut size compared with the mutant (*P*=0.051; Fig. 6A,E), clearing of luminal bacterial plaques, and reduced intestinal leukocyte infiltration as seen by decreased *mpo* expression and *lyzc:egfp* punctates (*P*=0.0006; Fig. 6B–E). The similar response to anti-inflammatory agents in *hi559* larvae and to anti-inflammatory treatment in humans in reducing leukocyte infiltration suggests a conserved mode of action. Intriguingly, the antibiotic and anti-inflammatory treatments did not significantly alleviate GCs, autophagy and apoptosis of the *hi559* intestinal mucosa (*P*=0.527, 0.59, 0.298, respectively; Fig. 6C,E), suggesting that ER stress is a precursor to bacterial overgrowth and inflammation. Subsequently, inflammation might then further exacerbate GI pathology. Hence, we hypothesize that inflammation is a downstream pathological event of ER stress and that relieving ER stress in *hi559* larvae would rescue the GI inflammation.

**Fig. 7.**
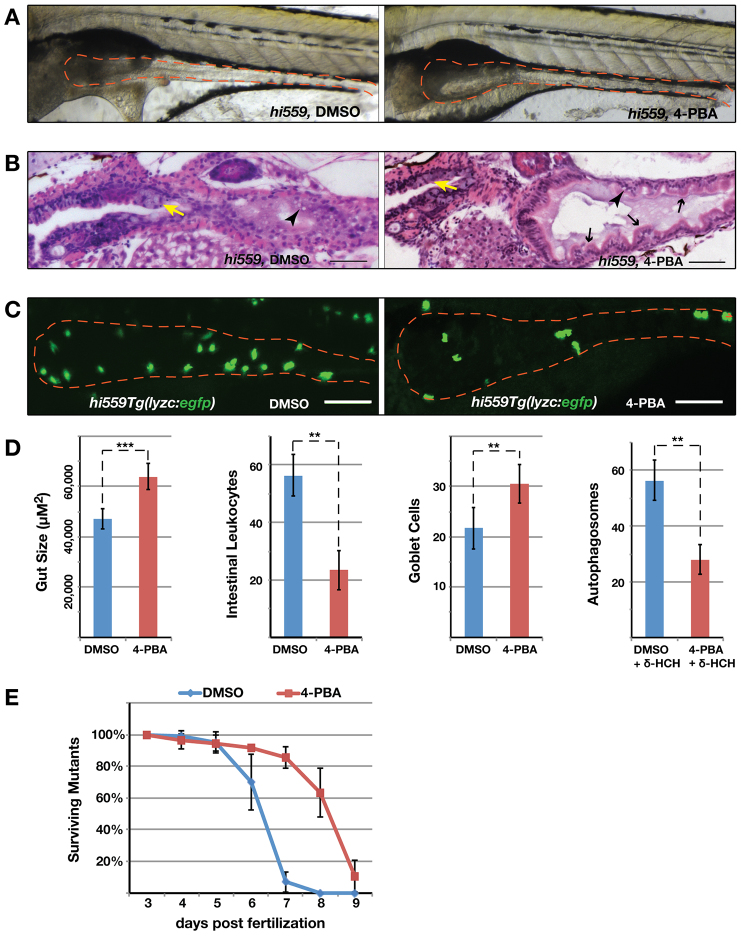
**Chemical chaperone 4-PBA rescues the *hi559* GI phenotype.** (A) Bright-field image of DMSO- and 4-PBA-treated *hi559* larvae at 6.5 dpf, showing amelioration of gross intestinal structure (red outline). (B) H&E-stained sagittal sections shows improved villous architecture (black arrows), GCs (yellow arrows) and reduction in mucosal necrosis (arrowheads) in 4-PBA-treated larvae. (C) Confocal projection of *hi559Tg(lyzc:egfp)* larval intestine (red outline) showing reduction in intestinal leukocyte infiltration in 4-PBA-treated larvae. (D) Bar charts of *hi559* larval gut size (*n*=15), intestinal leukocyte counts in *hi559Tg(lyzc:egfp)* larvae (*n*=12) and intestinal autophagosomes (*lc3:gfp* punctates) in δ-HCH-treated wild-type *Tg(lc3:gfp)* larvae (*n*=7) exposed to DMSO or 4-PBA. (E) Survival curve of *hi559* larvae treated with DMSO or 4-PBA from 3 dpf through to 9 dpf. Error bars indicate s.d. **P<0.01, ***P<0.001. Scale bars: 20 μm (B); 50 μm (C).

Chemical chaperones enhance the protein folding and adaptive capacity of the ER and thus act as a potent suppressor of ER stress. As a proof of principle, we investigated whether the *hi559* larvae are responsive to chemical chaperones that might alleviate ER stress. Phenylbutyric acid (PBA) is a small chemical chaperone and a well-established drug proven to reduce ER stress in both *in vivo* and *in vitro* studies (Ozcan et al., 2006). Prolonged exposure of *hi559* larvae to 4-PBA ameliorated the intestinal phenotype, showing significant increase in gut size (*P*=0.0006; Fig. 7A,D), improvement in intestinal villous architecture (Fig. 7B), mitigation of inflammation with a significant reduction of intestinal leukocyte infiltration (*P*=0.0037; Fig. 7C,D), and increased survival of GCs (*P*=0.0031; Fig. 7D). Exposure of 4-PBA in δ-HCH-treated *Tg(lc3:gfp)* larvae showed reduction of IEC autophagosomes (*P*=0.0028; Fig. 7D), suggesting that alleviating ER stress can potentially reduce autophagy in PI-deficient larvae. Notably, the alleviation of *hi559* GI phenotype by 4-PBA also resulted in improved survival of the mutant larvae (*P*=0.0008; Fig. 7E). Taken together, these results strongly support a feedback model in which unresolved ER stress initially triggers inflammation, which in turn can further worsen ER stress, exacerbating the GI pathology.

## DISCUSSION

Using zebrafish genetic and pharmacological models, this study demonstrates that intracellular PI synthesis plays a vital role in maintaining physiological homeostasis and integrity of intestinal mucosa. Analysis of *hi559* mutants has proven that loss of Cdipt function abrogates *de novo* PI synthesis, resulting in multiple GI pathologies. The *cdipt* mutant intestine fails to maintain its integrity and villous architecture as IECs exhibit reduced proliferation, reduced columnar shape, and degeneration at later larval stages. PI signaling has been linked to both cytokinesis and specification of apicobasal polarity in epithelial organs (Janetopoulos et al., 2005; Janetopoulos and Devreotes, 2006; Comer and Parent, 2007). Because IECs are rapidly proliferating at larval stages (Fig. 3A), they might require increased levels of *de novo* PI synthesis. Thus, despite maternal deposits of PI within the embryo itself (Thakur et al., 2011), these PI pools might not satisfy the specific needs of IECs in the *hi559* larval intestine, leading to aberrant cytological architecture and a decrease in proliferation.

Loss of *de novo* PI synthesis might impede various cellular functions; however, our data suggest a primary role for secretory pathways in the development of the *hi559* phenotype. Highly secretory cells of the digestive system of Cdipt-deficient larvae exhibit ER-stress-associated cytopathology, such as extensive disruption of the ER-Golgi complex and aggregates of electron-dense granules within the ER-Golgi lumen, suggesting protein accumulation consistent with pathological ER stress (Fig. 4). Furthermore, the observed diminution of mucus secretory functions of GCs and the aberrant ER-Golgi architecture of enteroendocrine and pancreatic cells clearly implicate a secretory defect in the *hi559* digestive system. A mutation in *sec13*, which is primarily involved in protein trafficking from ER to Golgi, causes similar ER disruption, cell-cycle arrest, and cell death phenotypes in zebrafish (Niu et al., 2012), thus substantiating the hypothesis that a secretory defect might be linked to the persistent ER stress pathology. Given the role of PI metabolism and its phosphorylated derivatives (PIPs) in vesicular trafficking at ER exit sites and within the secretory pathway (Hama et al., 1999; Blumental-Perry et al., 2006; Yakir-Tamang and Gerst, 2009), it is likely that the tissue-specific expression of *cdipt* reflects a requirement for *de novo* intracellular PI pools by IECs to support the secretory function of intestinal cells, which could make them particularly vulnerable to ER stress. Therefore, specific intracellular PI signaling components might be intrinsic regulators of ER stress and UPR (Jesch et al., 2006), and we are pursuing their identification.

We observed abnormal cell death in the mutant intestine, predominantly of GCs by apoptosis. Interestingly, loss of GCs has been linked to human IBD pathogenesis (Xavier and Podolsky, 2007). Chronic ER stress and UPR can lead to cell death through different pathways, such as upregulation of apoptotic factors, including *casp3* or *ddit3/chop*, which are found to be upregulated in *hi559* larvae (Thakur et al., 2011), or via release of Ca^2+^ from the ER, which perturbs mitochondria and triggers oxidative-stress-induced cell death and inflammation (Kim et al., 2008; Lin et al., 2008). Additionally, ER stress is linked to increased autophagy (Yorimitsu et al., 2006), which is clearly evident in *hi559* GI cells. These mechanisms could plausibly explain the increased apoptosis, mitochondrial damage and autophagy in *hi559* GI cells, which collectively manifest in GC loss and intestinal dysfunction.

Increased inflammation in the *hi559* intestine could be the result of at least two complementary mechanisms. Dysfunction and apoptosis of GCs and other IECs might result in reduced secretion of antimicrobial mucus and peptides. This loss would facilitate bacterial overgrowth, resulting in an inflammatory response to the increased intraluminal microbial load. Separately, in *hi559* larvae, we observed the reported ER-UPR dependent dysregulation of NF-κB and pro-inflammatory interleukin signaling (Fig. 3 and supplementary material Fig. S4), which probably contributes to inflammation (Yamazaki et al., 2009; Kaser and Blumberg, 2010). This could set up a positive feedback loop in which bacterial overgrowth causes upregulation in the synthesis of secreted antimicrobial peptides and mucus, thus adding to the stress of the ER-Golgi secretory complex and further straining the intestinal mucosa. Additional studies using a recently developed NF-κB reporter line (Kanther et al., 2011), reared in a gnotobiotic environment, could help to dissect the complex interplay of gut microbiota, NF-κB signaling and ER stress in intestinal inflammation.

Although inflammation itself might contribute to ER stress, multiple lines of evidence in our study support a model in which ER stress initially triggers the development of necro-inflammatory injury. Temporal analysis of the *hi559* ultrastructural pathology showed that hallmarks of ER stress occur prior to the onset of gross intestinal inflammatory pathology. Furthermore, pharmacological induction of ER stress in wild-type larvae using tunicamycin resulted in apoptosis of intestinal GCs, bacterial overgrowth and increased inflammation similar to that seen in *hi559* larvae. Lastly, co-administration of antibiotics and anti-inflammatory drugs suppressed bacterial overgrowth and mucosal inflammation, but failed to alleviate the ER-stress-associated autophagy and necro-apoptosis (Fig. 6). Interestingly, treatment with the chemical chaperone 4-PBA, a known alleviator of ER stress, resulted in amelioration of the mucosal inflammation and increased survival of *hi559* larvae (Fig. 7). Collectively, these results strongly suggest that bacterial overgrowth and inflammation do not directly cause ER stress, but result from it and exacerbate the ER-stress-induced pathology in our model.

This finding has important implications for the treatment of many human GI diseases. It suggests that pharmacologic manipulation of the ER stress pathway might be a novel treatment paradigm for particular GI diseases (such as IBD) and other disparate diseases (including cancer) that have been linked with chronic inflammation. Phospholipids are believed to have potential anti-inflammatory roles and can suppress activation of pro-inflammatory cells *in vivo*. Phosphatidylserine has been shown to inhibit macrophage activation (Gilbreath et al., 1985), and administration of phosphatidylcholine prevented stricture formation in a rat model of colitis (Mourelle et al., 1996). Interestingly, the PI3-K subunit p110δ was shown to play a vital role in maintaining mucosal homeostasis. Its inactivation caused defects in B and T cell signaling and in bactericidal activity, resulting in chronic colitis in mice (Uno et al., 2010). Recently, PI itself has been shown to inhibit T cell proliferation and function, implicating it as a novel physiological immune suppressant (van Dieren et al., 2011). The μ-opioid receptor ligand DALDA, a compound that might activate PI3-K signaling, has been shown to protect glafenine-induced intestinal injury in zebrafish by ameliorating ER stress (Goldsmith et al., 2013). We hypothesize that PI exerts its anti-inflammatory function via its ability to alleviate pathological ER stress.

This study provides the first evidence linking PI synthase to ER-stress-mediated GI pathologies, including bacterial overgrowth, mucosal apoptosis and inflammation, that are reminiscent of human IBD. In addition to genes regulating the immune system, mutations in genes affecting epithelial ER stress and function have been associated with IBD risk factors (Khor et al., 2011). Because ER homeostasis and inflammatory pathways appear to be conserved between zebrafish and human, investigating the mechanisms of ER stress in the zebrafish might reveal novel markers for IBD treatment. Currently, metabolically stabilized PI-derivative analogs and ER-stress-modulating compounds are being tested for their physiological relevance (Kim et al., 2008; He et al., 2011). Within this context, the *cdipt* mutants could provide an excellent platform for preclinical *in vivo* whole-organism studies evaluating the therapeutic potential of such compounds in ameliorating epithelial injury and inflammation.

## MATERIALS AND METHODS

### Zebrafish lines, embryo collection and genotyping

The zebrafish line *cdipt^hi559^* was isolated from a large-scale insertional mutagenesis screen (Amsterdam et al., 2004). Heterozygous and homozygous mutants were sorted by genotyping using PCR (Thakur et al., 2011). The *cdipt^lop^* mutant was isolated from a chemical mutagenesis screen (Murphy et al., 2011). The *cdipt^hi559/lop^trans*-heterozygotes were generated by crossing *hi559* heterozygotes with *cdipt^lop^*. Fish were maintained in accordance with the institutional animal care and use committee protocols.

### Development of *Tg(fabp2:egfp)* transgenic zebrafish

We used the regulatory region of the zebrafish *fatty acid binding protein 2, intestinal* (*fabp2*) to generate a transgenic zebrafish line. A 1.2-kb upstream fragment of the *fabp2* promoter was cloned into the plasmid vector *pEGFP*. The plasmids *pEGFP-fabp2* and *pTOL2 (pT2KxIG in)* were double-digested with *Bam*HI and *Xho*I. The linearized ~3.5-kb fragment from *pEGFP-fabp2* and the 6.8-kb fragment from *pTOL2* were ligated using the *T4 DNA* ligase. The linearized construct *pTOL2-EGFP-fabp2* was micro-injected into single-cell zebrafish embryos to obtain a germ-line transgene integration of *fabp2-TOL2-EGFP*. The founder fish were screened for the stable integration of the transgene, and subsequent transgenic fish generations were maintained.

### Live imaging of transgenic zebrafish

Double transgenic fish used in this study were generated by crossing *hi559* heterozygotes with *Tg(gut:gfp)* and *Tg(lyzc:egfp)* lines. The *Tg(gut:gfp)* transgenic zebrafish line expresses GFP throughout the digestive system and is used as a tool to analyze development of the GI tract and digestive organs (Field et al., 2003). The *Tg(lyzc:egfp)* transgenic line expresses enhanced green fluorescent protein (EGFP) under the regulatory regions of the zebrafish *lysozyme-C* (*lyzc*) gene, and is used to study infiltration of myeloid-derived inflammatory cells, representing a subset of macrophages and granulocytes (Hall et al., 2007; Hall et al., 2009). The *Tg(mpx:gfp)* transgenic line expresses GFP under the neutrophil-specific *myeloperoxidase* (*mpx*, also known as *mpo*) promoter and is used effectively to analyze intravital inflammatory response *in vivo* in zebrafish larvae (Renshaw et al., 2006). The *Tg(lc3:gfp)* transgenic line expresses GFP-fused Lc3 (GFP-Lc3), which can be visualized *in vivo* to monitor autophagy as Lc3 specifically labels the growing phagophores and completed autophagosomes (Kabeya et al., 2000; He et al., 2009; He and Klionsky, 2010).

Live imaging of zebrafish larvae was done by brightfield or fluorescent microscopy (Leica or Zeiss Axiovert). Confocal imaging was performed using a laser scanning confocal microscope (Leica or Zeiss), and the acquired images were analyzed using ImageJ (NIH, Bethesda, MD). The GFP intensity and puncta were quantified to assess leukocytes and autophagosomes in the GI tract of the respective transgenic larvae (*n*≥12).

### Whole-mount staining

For Nile Red staining, larvae were treated with 10 ng/ml Nile Red in E3 medium, starting at 3 dpf. The size and morphology of the gut lumen was assessed at different stages by observing Nile Red incorporation using fluorescent microscopy (Leica). Cy3-SA labeling and whole-mount *in situ* hybridization were performed as described previously (Sadler et al., 2005; Stuckenholz et al., 2009). Quantitative analyses of gut size were performed by ImageJ analyses of GFP-positive intestinal area using the *Tg(gut:gfp)* or *Tg(fabp2-egfp)* transgenic fish.

### Quantification of histological data

Histological sectioning and hematoxylin and eosin (H&E) staining were performed as described previously (Thakur et al., 2011). The IEC morphology, villous architecture and histological evidence of intraluminal bacteria were assessed by microscopic examination (Zeiss Axiovert) of at least ten alternate H&E-stained sagittal sections (5 μM), each representing larvae from wild type and mutants (*n*≥15) and larvae from DMSO control and drug treatment groups (*n*≥7). This allowed us to cover the analyses of histological features of the entire GI tract. For GC enumeration, PAS- and H&E-stained sections prepared at various time points during larval growth were imaged, and the total numbers of IECs and PAS-positive cells were determined for at least 12 alternate sections (4 μM) representing at least eight different larvae each from wild-type, mutant, DMSO and drug treatment groups. Phenotypically mature GCs were assessed according to the intensity of staining, the size of the apical region, the location in the intestinal epithelium and morphological appearance.

### Immunofluorescence assay

To assess the differential expression of Hspa5, sagittal cryosections (8 μM) through the entire GI tract of wild-type and mutant larvae were used for fluorescent immunohistochemistry using anti-HSPA5 primary antibody (Sigma) and FITC-conjugated anti-rabbit IgG secondary antibody (Sigma) and counterstained with DAPI (Sigma) for nuclear staining. Fluorescent image acquisition was performed using a confocal microscope (Zeiss) followed by analyses using ImageJ.

### TEM data analyses

TEM was performed in the EM facility at the Center for Biological Imaging, University of Pittsburgh. For semithin sections, the epoxy resin-embedded larvae were transverse sectioned (350 nm) and stained with toluidine blue. At least ten ultrathin sections (70 nm) were collected, corresponding to the esophageal and intestinal region, for TEM staining and analyses. Sectioning depth from the beginning of the tissue as reference point and visualization of toluidine blue stained sections at regular intervals allowed us to select TEM sections from the same area of the tissue for wild type and mutants. The number of mitochondria, autophagosomes and lysosomes were counted from a set field of specific magnifications facilitating observation of GI cells and represented as numbers per field of observation. GI cells containing double or multi-membrane autophagosomes were considered positive and counted manually. Data were presented as percentage of IECs with autophagy in each field of observation. Mitochondrial counts were presented as total number of mitochondria per IEC.

### Reverse transcriptase PCR

Total RNA from the micro-dissected GI tissue (*n*=5) was isolated using RNA purification kit (Stratagene). cDNA was prepared by reverse transcription using Superscript II (Invitrogen). RT-PCR to detect *xbp1* splicing was performed as described previously (Cinaroglu et al., 2011).

### Analyses of intestinal bacteria

Quantification of intestinal bacterial density was adapted from previously described methods with applicable modifications (Oehlers et al., 2011b). Zebrafish larvae (6 dpf) were euthanized with tricaine (MS-222, Sigma) and washed three times with sterile PBS containing 0.1% Tween to remove non-adherent or loosely attached surface bacteria. Individual guts from each larva were micro-dissected using disposable sterile needles (*n*=3 for each genotype or treatment group) and the isolated gut tissues were washed three times with sterile PBS followed by homogenization with 500 μl PBS in sterile microfuge tubes with disposable microfuge pestles. Serial log_10_ dilutions of the homogenates were plated on LB agar plates and incubated overnight at 28.5°C. Intestinal bacterial density was enumerated based on total colony forming units (cfu) per individual gut.

### Cell proliferation and apoptosis

Cell proliferation was estimated by *in vivo* labeling with 5-bromo-2 -deoxy-uridine (BrdU, Roche) and apoptosis was quantified by TUNEL assay on histological sections using the ApopTag peroxidase kit (Chemicon). Larvae (4, 5 and 6 dpf) were incubated in E3 with 10 mM BrdU for 6 h at 28.5°C and fixed in 4% PFA overnight. Incorporated BrdU was detected with an anti-BrdU antibody (Amersham) and visualized with peroxidase substrate kit (Vector). Quantification of proliferating and apoptotic cells was represented in percentages of relative proportion of BrdU-positive and TUNEL-positive IECs to the total number of IECs in at least ten alternate sagittal sections (5 μm) representing the entire GI tract of at least eight different larvae.

### Pharmacological assays

Tunicamycin is known to prevent N-linked glycosylation of proteins, affecting their proper folding and accumulation in the ER, thus inducing ER stress in eukaryotic cells (Yoshida, 2007). The tunicamycin (Calbiochem, EMD Biosciences) treatment assay in larval zebrafish was optimized using the methods described in our previous study (Thakur et al., 2011).

δ-Hexachlorocyclohexane (δ-HCH), a compound with similar configuration to myo-inositol, inhibits PI synthesis by affecting the incorporation of myo-inositol into PI (Hoffmann et al., 1980; Thakur et al., 2011). Wild-type, *Tg(gut:gfp)*, *Tg(lyzc:egfp)* or *Tg(lc3:gfp)* larvae were treated with 5 μM δ-HCH (Sigma) from 3.5 to 6 dpf and analyzed at 6 dpf.

For antibiotic treatment, zebrafish larvae were exposed to a cocktail of antibiotics from 3.5 to 6 dpf. Antibiotics consisted of ampicillin (Sigma, 100 μg/ml final concentration), kanamycin (Sigma, 5 μg/ml final concentration), and penicillin-streptomycin pre-mix (Invitrogen, 100 units/ml penicillin and 100 μg/ml streptomycin) in E3 media. Anti-inflammatory drugs consisted of 5-aminosalysilic acid (ASA; Sigma, 50 μg/ml) and 6-α-methylprednisolone (Sigma, 25 μg/ml) dissolved in 0.05% DMSO v/v in E3 media.

For chemical chaperone treatment, sodium 4-PBA (Sigma) was dissolved in E3 water and the fish exposed to various dosages of 4-PBA to optimize a nontoxic dosage that did not cause any developmental defects. A final concentration of 50 μM 4-PBA (from 3.5 dpf until 9 dpf) was used for the rescue experiments in this study. For control groups, larvae were treated with equivalent concentration of DMSO alone. The drug treatments were performed in 12-well plates containing 15 larvae each from three different biological clutches.

### Statistics

Data are representative of larvae from at least three different biological clutches. Statistical significance was calculated using a two-tailed Student’s *t*-test; *P* values of less than 0.05 were considered significant. The results are expressed as means, and standard deviations are indicted by error bars in the figures.

### Pathway analyses

Gene expression and pathway analyses were performed as described previously using our microarray data deposited with GEO (GSE17711) (Stuckenholz et al., 2009; Thakur et al., 2011). We used Ingenuity’s pathway analysis tool (IPA; http://www.ingenuity.com) and the Gene Set Enrichment Analysis tool (GSEA; http://www.broad.mit.edu/gsea/) (Subramanian et al., 2005) to decipher the dysregulated pathways in the mutants. A *P* value of less than 0.05 (*n*=3) after adjusting for false discovery rate was considered significant.

## Supplementary Material

Supplementary Material

## References

[b1-0070093] Abraham C. Cho J. H. (2009). Inflammatory bowel disease. N. Engl. J. Med. 361, 2066–2078 19923578 10.1056/NEJMra0804647 PMC3491806

[b2-0070093] Amsterdam A. Nissen R. M. Sun Z. Swindell E. C. Farrington S. Hopkins N. (2004). Identification of 315 genes essential for early zebrafish development. Proc. Natl. Acad. Sci. USA 101, 12792–12797 15256591 10.1073/pnas.0403929101 PMC516474

[b3-0070093] Anderson C. A. Boucher G. Lees C. W. Franke A. D’Amato M. Taylor K. D. Lee J. C. Goyette P. Imielinski M. Latiano A. (2011). Meta-analysis identifies 29 additional ulcerative colitis risk loci, increasing the number of confirmed associations to 47. Nat. Genet. 43, 246–252 21297633 10.1038/ng.764 PMC3084597

[b4-0070093] Balla T. Szentpetery Z. Kim Y. J. (2009). Phosphoinositide signaling: new tools and insights. Physiology (Bethesda) 24, 231–244 19675354 10.1152/physiol.00014.2009 PMC3126675

[b5-0070093] Blumental-Perry A. Haney C. J. Weixel K. M. Watkins S. C. Weisz O. A. Aridor M. (2006). Phosphatidylinositol 4-phosphate formation at ER exit sites regulates ER export. Dev. Cell 11, 671–682 17084359 10.1016/j.devcel.2006.09.001

[b6-0070093] Bogaert S. De Vos M. Olievier K. Peeters H. Elewaut D. Lambrecht B. Pouliot P. Laukens D. (2011). Involvement of endoplasmic reticulum stress in inflammatory bowel disease: a different implication for colonic and ileal disease? PLoS ONE 6, e25589 22028783 10.1371/journal.pone.0025589 PMC3196494

[b7-0070093] Calfon M. Zeng H. Urano F. Till J. H. Hubbard S. R. Harding H. P. Clark S. G. Ron D. (2002). IRE1 couples endoplasmic reticulum load to secretory capacity by processing the XBP-1 mRNA. Nature 415, 92–96 11780124 10.1038/415092a

[b8-0070093] Cinaroglu A. Gao C. Imrie D. Sadler K. C. (2011). Activating transcription factor 6 plays protective and pathological roles in steatosis due to endoplasmic reticulum stress in zebrafish. Hepatology 54, 495–508 21538441 10.1002/hep.24396 PMC3145024

[b9-0070093] Comer F. I. Parent C. A. (2007). Phosphoinositides specify polarity during epithelial organ development. Cell 128, 239–240 17254962 10.1016/j.cell.2007.01.010

[b10-0070093] Deng J. Lu P. D. Zhang Y. Scheuner D. Kaufman R. J. Sonenberg N. Harding H. P. Ron D. (2004). Translational repression mediates activation of nuclear factor kappa B by phosphorylated translation initiation factor 2. Mol. Cell. Biol. 24, 10161–10168 15542827 10.1128/MCB.24.23.10161-10168.2004 PMC529034

[b11-0070093] English A. R. Voeltz G. K. (2013). Rab10 GTPase regulates ER dynamics and morphology. Nat. Cell Biol. 15, 169–178 23263280 10.1038/ncb2647 PMC3582403

[b12-0070093] Field H. A. Ober E. A. Roeser T. Stainier D. Y. (2003). Formation of the digestive system in zebrafish. I. Liver morphogenesis. Dev. Biol. 253, 279–290 12645931 10.1016/s0012-1606(02)00017-9

[b13-0070093] Fleming A. Jankowski J. Goldsmith P. (2010). In vivo analysis of gut function and disease changes in a zebrafish larvae model of inflammatory bowel disease: a feasibility study. Inflamm. Bowel Dis. 16, 1162–1172 20128011 10.1002/ibd.21200

[b14-0070093] Franke A. McGovern D. P. Barrett J. C. Wang K. Radford-Smith G. L. Ahmad T. Lees C. W. Balschun T. Lee J. Roberts R. (2010). Genome-wide meta-analysis increases to 71 the number of confirmed Crohn’s disease susceptibility loci. Nat. Genet. 42, 1118–1125 21102463 10.1038/ng.717 PMC3299551

[b15-0070093] Gilbreath M. J. Nacy C. A. Hoover D. L. Alving C. R. Swartz G. M. Jr Meltzer M. S. (1985). Macrophage activation for microbicidal activity against Leishmania major: inhibition of lymphokine activation by phosphatidylcholine-phosphatidylserine liposomes. J. Immunol. 134, 3420–3425 3980997

[b16-0070093] Goldsmith J. R. Cocchiaro J. L. Rawls J. F. Jobin C. (2013). Glafenine-induced intestinal injury in zebrafish is ameliorated by μ-opioid signaling via enhancement of Atf6-dependent cellular stress responses. Dis. Model. Mech. 6, 146–159 22917923 10.1242/dmm.009852 PMC3529347

[b17-0070093] Hall C. Flores M. V. Storm T. Crosier K. Crosier P. (2007). The zebrafish lysozyme C promoter drives myeloid-specific expression in transgenic fish. BMC Dev. Biol. 7, 42 17477879 10.1186/1471-213X-7-42 PMC1877083

[b18-0070093] Hall C. Flores M. V. Crosier K. Crosier P. (2009). Live cell imaging of zebrafish leukocytes. Methods Mol. Biol. 546, 255–271 19378109 10.1007/978-1-60327-977-2_16

[b19-0070093] Hama H. Schnieders E. A. Thorner J. Takemoto J. Y. DeWald D. B. (1999). Direct involvement of phosphatidylinositol 4-phosphate in secretion in the yeast Saccharomyces cerevisiae. J. Biol. Chem. 274, 34294–34300 10567405 10.1074/jbc.274.48.34294

[b20-0070093] He C. Klionsky D. J. (2010). Analyzing autophagy in zebrafish. Autophagy 6, 642–644 20495344 10.4161/auto.6.5.12092 PMC3654832

[b21-0070093] He C. Bartholomew C. R. Zhou W. Klionsky D. J. (2009). Assaying autophagic activity in transgenic GFP-Lc3 and GFP-Gabarap zebrafish embryos. Autophagy 5, 520–526 19221467 10.4161/auto.5.4.7768 PMC2754832

[b22-0070093] He J. Gajewiak J. Scott J. L. Gong D. Ali M. Best M. D. Prestwich G. D. Stahelin R. V. Kutateladze T. G. (2011). Metabolically stabilized derivatives of phosphatidylinositol 4-phosphate: synthesis and applications. Chem. Biol. 18, 1312–1319 22035800 10.1016/j.chembiol.2011.07.022 PMC3205354

[b23-0070093] Heazlewood C. K. Cook M. C. Eri R. Price G. R. Tauro S. B. Taupin D. Thornton D. J. Png C. W. Crockford T. L. Cornall R. J. (2008). Aberrant mucin assembly in mice causes endoplasmic reticulum stress and spontaneous inflammation resembling ulcerative colitis. PLoS Med. 5, e54 18318598 10.1371/journal.pmed.0050054 PMC2270292

[b24-0070093] Henry M. C. Moss R. L. (2009). Necrotizing enterocolitis. Annu. Rev. Med. 60, 111–124 18817461 10.1146/annurev.med.60.050207.092824

[b25-0070093] Hoffmann R. Erzberger P. Frank W. Ristow H. J. (1980). Increased phosphatidylinositol synthesis in rat embryo fibroblasts after growth stimulation and its inhibition by delta-hexachlorocyclohexane. Biochim. Biophys. Acta 618, 282–292 6155147 10.1016/0005-2760(80)90034-x

[b26-0070093] Hotamisligil G. S. (2010). Endoplasmic reticulum stress and the inflammatory basis of metabolic disease. Cell 140, 900–917 20303879 10.1016/j.cell.2010.02.034 PMC2887297

[b27-0070093] Hu P. Han Z. Couvillon A. D. Kaufman R. J. Exton J. H. (2006). Autocrine tumor necrosis factor alpha links endoplasmic reticulum stress to the membrane death receptor pathway through IRE1alpha-mediated NF-kappaB activation and down-regulation of TRAF2 expression. Mol. Cell. Biol. 26, 3071–3084 16581782 10.1128/MCB.26.8.3071-3084.2006 PMC1446932

[b28-0070093] Janetopoulos C. Devreotes P. (2006). Phosphoinositide signaling plays a key role in cytokinesis. J. Cell Biol. 174, 485–490 16908667 10.1083/jcb.200603156 PMC2064254

[b29-0070093] Janetopoulos C. Borleis J. Vazquez F. Iijima M. Devreotes P. (2005). Temporal and spatial regulation of phosphoinositide signaling mediates cytokinesis. Dev. Cell 8, 467–477 15809030 10.1016/j.devcel.2005.02.010

[b30-0070093] Jesch S. A. Liu P. Zhao X. Wells M. T. Henry S. A. (2006). Multiple endoplasmic reticulum-to-nucleus signaling pathways coordinate phospholipid metabolism with gene expression by distinct mechanisms. J. Biol. Chem. 281, 24070–24083 16777852 10.1074/jbc.M604541200

[b31-0070093] Kabeya Y. Mizushima N. Ueno T. Yamamoto A. Kirisako T. Noda T. Kominami E. Ohsumi Y. Yoshimori T. (2000). LC3, a mammalian homologue of yeast Apg8p, is localized in autophagosome membranes after processing. EMBO J. 19, 5720–5728 11060023 10.1093/emboj/19.21.5720 PMC305793

[b32-0070093] Kanther M. Sun X. Mühlbauer M. Mackey L. C. Flynn E. J. 3rd Bagnat M. Jobin C. Rawls J. F. (2011). Microbial colonization induces dynamic temporal and spatial patterns of NF-κB activation in the zebrafish digestive tract. Gastroenterology 141, 197–207 21439961 10.1053/j.gastro.2011.03.042 PMC3164861

[b33-0070093] Kaser A. Blumberg R. S. (2009). Endoplasmic reticulum stress in the intestinal epithelium and inflammatory bowel disease. Semin. Immunol. 21, 156–163 19237300 10.1016/j.smim.2009.01.001 PMC4736746

[b34-0070093] Kaser A. Blumberg R. S. (2010). Endoplasmic reticulum stress and intestinal inflammation. Mucosal Immunol. 3, 11–16 19865077 10.1038/mi.2009.122 PMC4592136

[b35-0070093] Kaser A. Lee A. H. Franke A. Glickman J. N. Zeissig S. Tilg H. Nieuwenhuis E. E. Higgins D. E. Schreiber S. Glimcher L. H. (2008). XBP1 links ER stress to intestinal inflammation and confers genetic risk for human inflammatory bowel disease. Cell 134, 743–756 18775308 10.1016/j.cell.2008.07.021 PMC2586148

[b36-0070093] Khor B. Gardet A. Xavier R. J. (2011). Genetics and pathogenesis of inflammatory bowel disease. Nature 474, 307–317 21677747 10.1038/nature10209 PMC3204665

[b37-0070093] Kim I. Xu W. Reed J. C. (2008). Cell death and endoplasmic reticulum stress: disease relevance and therapeutic opportunities. Nat. Rev. Drug Discov. 7, 1013–1030 19043451 10.1038/nrd2755

[b38-0070093] Kim Y. J. Guzman-Hernandez M. L. Balla T. (2011). A highly dynamic ER-derived phosphatidylinositol-synthesizing organelle supplies phosphoinositides to cellular membranes. Dev. Cell 21, 813–824 22075145 10.1016/j.devcel.2011.09.005 PMC3235737

[b39-0070093] Lin J. H. Walter P. Yen T. S. (2008). Endoplasmic reticulum stress in disease pathogenesis. Annu. Rev. Pathol. 3, 399–425 18039139 10.1146/annurev.pathmechdis.3.121806.151434 PMC3653419

[b40-0070093] Marciniak S. J. Ron D. (2006). Endoplasmic reticulum stress signaling in disease. Physiol. Rev. 86, 1133–1149 17015486 10.1152/physrev.00015.2006

[b41-0070093] Mourelle M. Guarner F. Malagelada J. R. (1996). Polyunsaturated phosphatidylcholine prevents stricture formation in a rat model of colitis. Gastroenterology 110, 1093–1097 8612998 10.1053/gast.1996.v110.pm8612998

[b42-0070093] Murphy T. R. Vihtelic T. S. Ile K. E. Watson C. T. Willer G. B. Gregg R. G. Bankaitis V. A. Hyde D. R. (2011). Phosphatidylinositol synthase is required for lens structural integrity and photoreceptor cell survival in the zebrafish eye. Exp. Eye Res. 93, 460–474 21722635 10.1016/j.exer.2011.06.010 PMC3206183

[b43-0070093] Ng A. N. de Jong-Curtain T. A. Mawdsley D. J. White S. J. Shin J. Appel B. Dong P. D. Stainier D. Y. Heath J. K. (2005). Formation of the digestive system in zebrafish: III. Intestinal epithelium morphogenesis. Dev. Biol. 286, 114–135 16125164 10.1016/j.ydbio.2005.07.013

[b44-0070093] Niu X. Gao C. Jan Lo L. Luo Y. Meng C. Hong J. Hong W. Peng J. (2012). Sec13 safeguards the integrity of the endoplasmic reticulum and organogenesis of the digestive system in zebrafish. Dev. Biol. 367, 197–207 22609279 10.1016/j.ydbio.2012.05.004

[b45-0070093] Oehlers S. H. Flores M. V. Chen T. Hall C. J. Crosier K. E. Crosier P. S. (2011a). Topographical distribution of antimicrobial genes in the zebrafish intestine. Dev. Comp. Immunol. 35, 385–391 21093479 10.1016/j.dci.2010.11.008

[b46-0070093] Oehlers S. H. Flores M. V. Hall C. J. Swift S. Crosier K. E. Crosier P. S. (2011b). The inflammatory bowel disease (IBD) susceptibility genes NOD1 and NOD2 have conserved anti-bacterial roles in zebrafish. Dis. Model. Mech. 4, 832–841 21729873 10.1242/dmm.006122 PMC3209652

[b47-0070093] Ozcan U. Yilmaz E. Ozcan L. Furuhashi M. Vaillancourt E. Smith R. O. Görgün C. Z. Hotamisligil G. S. (2006). Chemical chaperones reduce ER stress and restore glucose homeostasis in a mouse model of type 2 diabetes. Science 313, 1137–1140 16931765 10.1126/science.1128294 PMC4741373

[b48-0070093] Perencevich M. Burakoff R. (2006). Use of antibiotics in the treatment of inflammatory bowel disease. Inflamm. Bowel Dis. 12, 651–664 16804403 10.1097/01.MIB.0000225330.38119.c7

[b49-0070093] Renshaw S. A. Loynes C. A. Trushell D. M. Elworthy S. Ingham P. W. Whyte M. K. (2006). A transgenic zebrafish model of neutrophilic inflammation. Blood 108, 3976–3978 16926288 10.1182/blood-2006-05-024075

[b50-0070093] Roeselers G. Mittge E. K. Stephens W. Z. Parichy D. M. Cavanaugh C. M. Guillemin K. Rawls J. F. (2011). Evidence for a core gut microbiota in the zebrafish. ISME J. 5, 1595–1608 21472014 10.1038/ismej.2011.38 PMC3176511

[b51-0070093] Ron D. Walter P. (2007). Signal integration in the endoplasmic reticulum unfolded protein response. Nat. Rev. Mol. Cell Biol. 8, 519–529 17565364 10.1038/nrm2199

[b52-0070093] Sadler K. C. Amsterdam A. Soroka C. Boyer J. Hopkins N. (2005). A genetic screen in zebrafish identifies the mutants vps18, nf2 and foie gras as models of liver disease. Development 132, 3561–3572 16000385 10.1242/dev.01918

[b53-0070093] Stuckenholz C. Ulanch P. E. Bahary N. (2004). From guts to brains: using zebrafish genetics to understand the innards of organogenesis. Curr. Top. Dev. Biol. 65, 47–82 15642379 10.1016/S0070-2153(04)65002-2

[b54-0070093] Stuckenholz C. Lu L. Thakur P. Kaminski N. Bahary N. (2009). FACS-assisted microarray profiling implicates novel genes and pathways in zebrafish gastrointestinal tract development. Gastroenterology 137, 1321–1332 19563808 10.1053/j.gastro.2009.06.050 PMC2785077

[b55-0070093] Subramanian A. Tamayo P. Mootha V. K. Mukherjee S. Ebert B. L. Gillette M. A. Paulovich A. Pomeroy S. L. Golub T. R. Lander E. S. (2005). Gene set enrichment analysis: a knowledge-based approach for interpreting genome-wide expression profiles. Proc. Natl. Acad. Sci. USA 102, 15545–15550 16199517 10.1073/pnas.0506580102 PMC1239896

[b56-0070093] Thakur P. C. Stuckenholz C. Rivera M. R. Davison J. M. Yao J. K. Amsterdam A. Sadler K. C. Bahary N. (2011). Lack of de novo phosphatidylinositol synthesis leads to endoplasmic reticulum stress and hepatic steatosis in cdipt-deficient zebrafish. Hepatology 54, 452–462 21488074 10.1002/hep.24349 PMC3140628

[b57-0070093] Uno J. K. Rao K. N. Matsuoka K. Sheikh S. Z. Kobayashi T. Li F. Steinbach E. C. Sepulveda A. R. Vanhaesebroeck B. Sartor R. B. (2010). Altered macrophage function contributes to colitis in mice defective in the phosphoinositide-3 kinase subunit p110delta. Gastroenterology 139, 1642–1653, e1–6 20637203 10.1053/j.gastro.2010.07.008 PMC2967619

[b58-0070093] van Dieren J. M. Simons-Oosterhuis Y. Raatgeep H. C. Lindenbergh-Kortleve D. J. Lambers M. E. van der Woude C. J. Kuipers E. J. Snoek G. T. Potman R. Hammad H. (2011). Anti-inflammatory actions of phosphatidylinositol. Eur. J. Immunol. 41, 1047–1057 21360703 10.1002/eji.201040899

[b59-0070093] Wallace K. N. Pack M. (2003). Unique and conserved aspects of gut development in zebrafish. Dev. Biol. 255, 12–29 12618131 10.1016/s0012-1606(02)00034-9

[b60-0070093] Xavier R. J. Podolsky D. K. (2007). Unravelling the pathogenesis of inflammatory bowel disease. Nature 448, 427–434 17653185 10.1038/nature06005

[b61-0070093] Yakir-Tamang L. Gerst J. E. (2009). A phosphatidylinositol-transfer protein and phosphatidylinositol-4-phosphate 5-kinase control Cdc42 to regulate the actin cytoskeleton and secretory pathway in yeast. Mol. Biol. Cell 20, 3583–3597 19477927 10.1091/mbc.E08-10-1073 PMC2719576

[b62-0070093] Yamazaki H. Hiramatsu N. Hayakawa K. Tagawa Y. Okamura M. Ogata R. Huang T. Nakajima S. Yao J. Paton A. W. (2009). Activation of the Akt-NF-kappaB pathway by subtilase cytotoxin through the ATF6 branch of the unfolded protein response. J. Immunol. 183, 1480–1487 19561103 10.4049/jimmunol.0900017 PMC2762936

[b63-0070093] Yorimitsu T. Nair U. Yang Z. Klionsky D. J. (2006). Endoplasmic reticulum stress triggers autophagy. J. Biol. Chem. 281, 30299–30304 16901900 10.1074/jbc.M607007200 PMC1828866

[b64-0070093] Yoshida H. (2007). ER stress and diseases. FEBS J. 274, 630–658 17288551 10.1111/j.1742-4658.2007.05639.x

