# Supplementary material for: Dysregulated phosphatidylinositol signaling promotes endoplasmic-reticulum-stress-mediated intestinal mucosal injury and inflammation in zebrafish
Source: Dis Model Mech. 2013 Oct 17;7(1):93–106. doi: 10.1242/dmm.012864 (PMC3882052; doi:10.1242/dmm.012864)
Supplement: Supplementary Material [file supp_7_1_93__index.html]

Dysregulated phosphatidylinositol signaling promotes endoplasmic-reticulum-stress-mediated intestinal mucosal injury and inflammation in zebrafish — Dysregulated phosphatidylinositol signaling promotes endoplasmic-reticulum-stress-mediated intestinal mucosal injury and inflammation in zebrafish — Supplementary Material 

# Dysregulated phosphatidylinositol signaling promotes endoplasmic-reticulum-stress-mediated intestinal mucosal injury and inflammation in zebrafish

## DMM012864 Supplementary Material

**Files in this Data Supplement:**

- **Supplementary Material PDF**
