# Supplementary material for: Dysregulated phosphatidylinositol signaling promotes endoplasmic-reticulum-stress-mediated intestinal mucosal injury and inflammation in zebrafish
Source: Dis Model Mech. 2013 Oct 17;7(1):93–106. doi: 10.1242/dmm.012864 (PMC3882052; doi:10.1242/dmm.012864)
Supplement: Supplementary Material [file supp_012864_DMM012864.pdf]

SUPPLEMENTARY FIGURE LEGENDS

Supplementary Fig. S1: The *cdipt*^{lop} (*lop*) and *cdipt*^{hi559} (*hi559*) mutants exhibit identical GI phenotype and fail to rescue each other in complementation assay. (**A**) Lateral view of ISH of intestine-specific markers *vil1* and *anxa2b* showing reduced expression in the *hi559* intestine at 5-dpf. (**B-F**) Morphology of intestine shown by bright-field images (*left panel*) and fluorescent images (*right panel*, Nile-Red staining shown by *white outline*). (**B**) GI morphology of *hi559* larvae at 6-dpf shows smaller intestine and lumen. (**C**) GI morphology of *lop* larvae shows smaller intestine and lumen at 8-dpf comparable to the 6-dpf *hi559* larvae. (**D**) *Cdipt*$^{hi559/lop}$ (*hi559* X *lop*) *trans*-heterozygote mutant larvae show smaller intestinal lumen at 7.5-dpf comparable to the 6-dpf *hi559* larvae. (**E**) Wild-type embryos were microinjected with either control morpholino or splice-blocking morpholino against *cdipt* at 1-cell stage and larvae were analyzed at 6-dpf (Thakur et al., 2011). *Cdipt* morphants show smaller intestinal structures replicating *hi559* phenotype. (**F**) Wild-type larvae were treated with PI synthase inhibitor δ-HCH shows smaller intestinal structures compared to DMSO treated larvae.

Supplementary Fig. S2: Abnormal goblet cells and mucosecretion in *hi559* intestinal mucosa. (**A**) Wild-type intestinal luminal area appears clear with mucinous granules and microvilli projections from IECs. The *hi559* lumen is often filled with detached cells (*black arrow*) and bacterial overgrowth at 5.5-dpf (*red arrow*). Nuclear condensation (*white arrow*) and cytoplasmic vacuoles (*arrowhead*) are apparent in the detached IEC, suggesting apoptosis. (**B**) At 5-dpf, wild-type mucinous GCs are mature with largely dense mucinous theca, whereas the *hi559* GCs have poorly developed theca with abnormal vesicles, cytoplasmic necrosis and bacterial invasion (*red arrow*). (**C-D**) PAS-staining (*pink*) showing the mucin-secreting mid-intestinal GCs (*arrow*) and secreted mucinous layer (*arrowhead*) of the mid-intestinal epithelium at (**C**) 5-dpf and (**D**) 6-dpf. The thick secreted mucinous layer, which is seen at the apical border of wild-type intestinal mucosa, is greatly diminished in *hi559* and the mucinous cells are disarranged and often detached into the lumen. (**E**) Bar-chart showing the number of mid-intestinal (Mi) GCs in wild-type and *hi559* intestine at 5 and 6-dpf (n=7, *P<0.05). Goblet cell

population is significantly depleted in *hi559* intestine at 6-dpf. Wild-type (WT) on *left* and *hi559* on *right panels*. Lu, intestinal lumen; Th, mucinous theca. Scale bar: A-B, 500 nm; C-D, 20 μM.

Supplementary Fig. S3: PI deficiency causes necro-inflammattion. (**A**) H&E-staining shows nicely arranged mature cylindrical IECs in wild-type with thick apical border, whereas *hi559* epithelium shows necro-inflammatory injury with vacuolated IECs, thinner apical border and necrotic foci (*arrow*). (**B**) Confocal projection of intestines of DMSO and δ-HCH-treated *Tg(lyzc:egfp)* larvae, showing leukocyte infiltration (*arrow*). Right panel shows bar-chart of leukocytes in DMSO and δ-HCH-treated wild-type intestines at 6-dpf (n=15, *P<0.05). Scale bar: A, 20 μM; B, 100 μM.

Supplementary Fig. S4: Activation of pro-inflammatory cascade in *hi559 larvae*. (**A**) Differential upregulation and the corresponding *P*-values of a set of pro-inflammatory gene features and APR complement cascade in *hi559* larvae. (**B**) GSEA enrichment plots and expression profile (shown by heat-map) of genes involved in the NF-κB pathway, suggesting their enrichment in *hi559* larvae (P=0.006).

Supplementary Fig. S5: Unresolved ER stress pathology of *hi559* GI tissues. (**A-B**) ISH shows elevated expression of the crucial ER stress-UPR markers *hspa5* (**A**) and *xbp1* (**B**) in the *hi559* GI tissue. (**C**) RT-PCR showing activation of *xbp1* by increased transcription of unspliced (*xbp1-u*) and spliced *xbp1* (*xbp1-s*) in 5-dpf *hi559* GI tissues. β-actin was used as RT-PCR control. (**D-E**) TEM images of 6-dpf wild-type (*left panels*) and *hi559* pancreatic cells (*right panels*). (**D**) Ultrastructure of the secretory granules-containing endocrine pancreatic cells shows increased ER luminal expansion and vacuolization, multi-lamellar autophagic bodies (*red outline*), and increased mitophagy (*red arrow*) in *hi559*. (**E**) ER luminal expansion is also apparent in the *hi559* pancreatic acinar cells containing zymogen granules. Er: endoplasmic reticulum; Sg, secretory granules; Zm; zymogen granules; Mt, mitochondria. Scale bars: D-E, 500 nm.

Supplementary Fig. S6: Tunicamycin-induced ER stress causes abnormal GCs and necro-inflammation in wild-type GI tissues. (**A**) Bright-field image of DMSO and tunicamycin-treated larvae showing intestinal morphology (*red outline*). (**B**) H&E-

stained sections of DMSO and tunicamycin-treated larval esophagus showing GC morphology (*arrow*). Note the condensed nuclei of *hi559* GCs, suggesting apoptosis, in tunicamycin-treated larvae. (**C**) H&E-stained sections of DMSO and tunicamycin-treated larval intestine showing necro-inflammatory injury (*pink outline*) and increased cytoplasmic vesicles (*arrow*) in the IECs of the tunicamycin-treated intestine. (**D**) Confocal projection of *Tg(mpx:gfp)* larval intestine shows increased neutrophil infiltration in tunicamycin-treated larvae. Corresponding bar-chart is shown in the *right panel* (***P*<0.01). TN, tunicamycin. Scale bars: B, C, 20 μM; D, 100 μM.

Supplementary Fig. S7: Suppression of inflammation by antibiotics or anti-inflammatory drugs. Larvae were treated with antibiotics (abx) or anti-inflammatory (ai) drugs from 3.5-dpf to 6-dpf. (**A**) ISH with *fabp2* shows no improvement of intestinal architecture in *hi559* larvae treated with abx or ai drugs. (**B**) ISH with *mpo* showing partial suppression of intestinal neutrophil infiltration (*arrows*) in abx-treated *hi559* larvae and complete suppression in ai-treated larvae. (**C**) H&E-staining shows clearing of luminal bacteria in abx-treated *hi559* larvae. Luminal bacteria (*asterisk*) are seen in ai-treated larvae. IEC necrosis and shedding (*arrow*) and vacuolation (*arrowhead*) are still apparent in both abx- and ai-treated larvae. (**E**) Bar-charts showing significant reduction of intestinal bacterial density (gut CFU) in abx-treated *hi559* larvae (***P*<0.01). Scale bars: C, 20 μM.

REFERENCES

Thakur, P. C., Stuckenholz, C., Rivera, M. R., Davison, J. M., Yao, J. K., Amsterdam, A., Sadler, K. C. and Bahary, N. (2011) 'Lack of de novo phosphatidylinositol synthesis leads to endoplasmic reticulum stress and hepatic steatosis in cdipt-deficient zebrafish', *Hepatology* 54(2): 452-62.

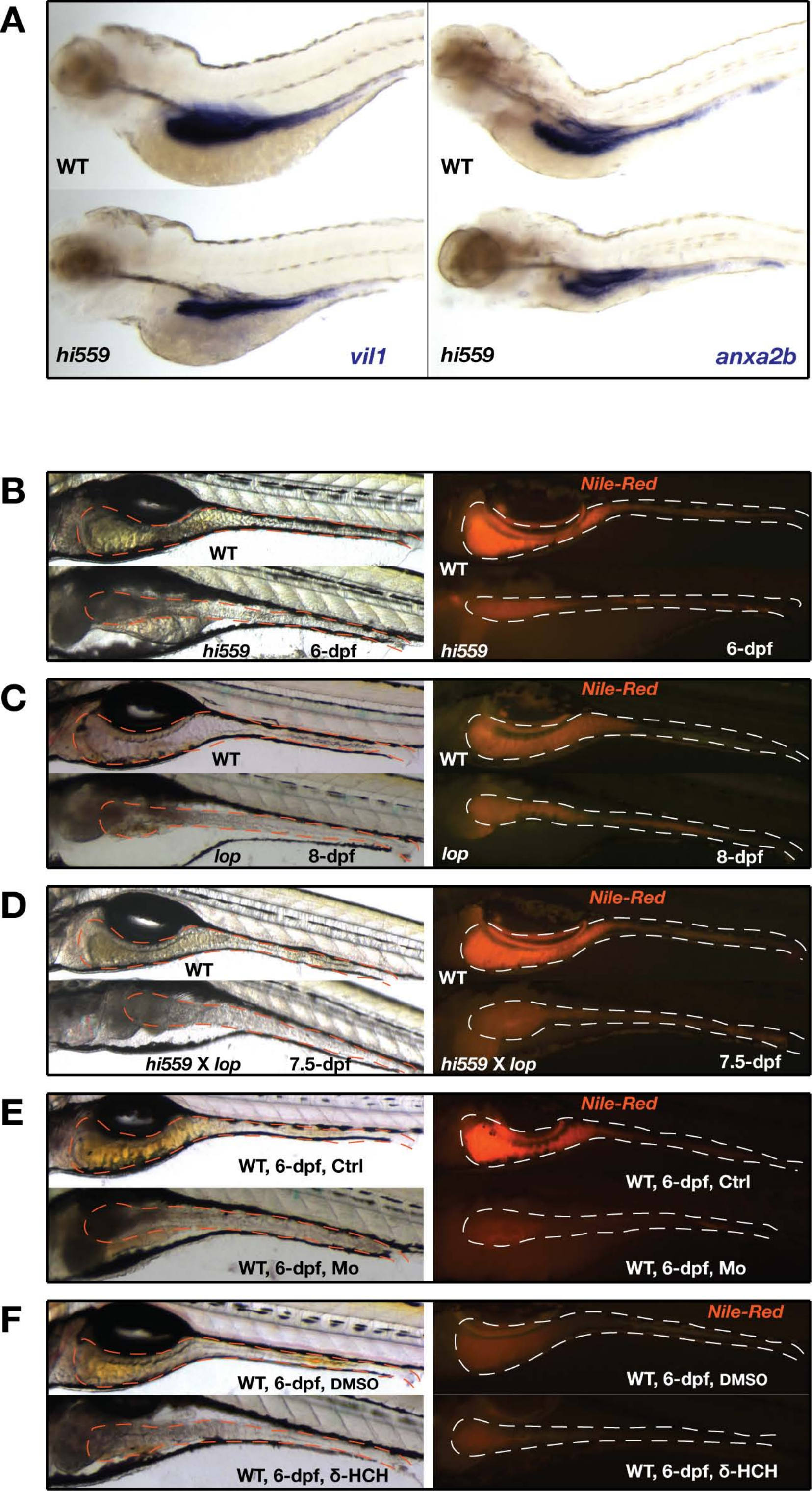

A
WT
WT
hi559
vil1
hi559
anxa2b
B
Nile-Red
WT
WT
hi559
6-dpf
hi559
6-dpf
C
Nile-Red
WT
WT
lop
8-dpf
lop
8-dpf
D
Nile-Red
WT
WT
hi559 X lop
7.5-dpf
hi559 X lop
7.5-dpf
E
Nile-Red
WT, 6-dpf, Ctrl
WT, 6-dpf, Ctrl
WT, 6-dpf, Mo
WT, 6-dpf, Mo
F
Nile-Red
WT, 6-dpf, DMSO
WT, 6-dpf, DMSO
WT, 6-dpf, δ-HCH
WT, 6-dpf, δ-HCH

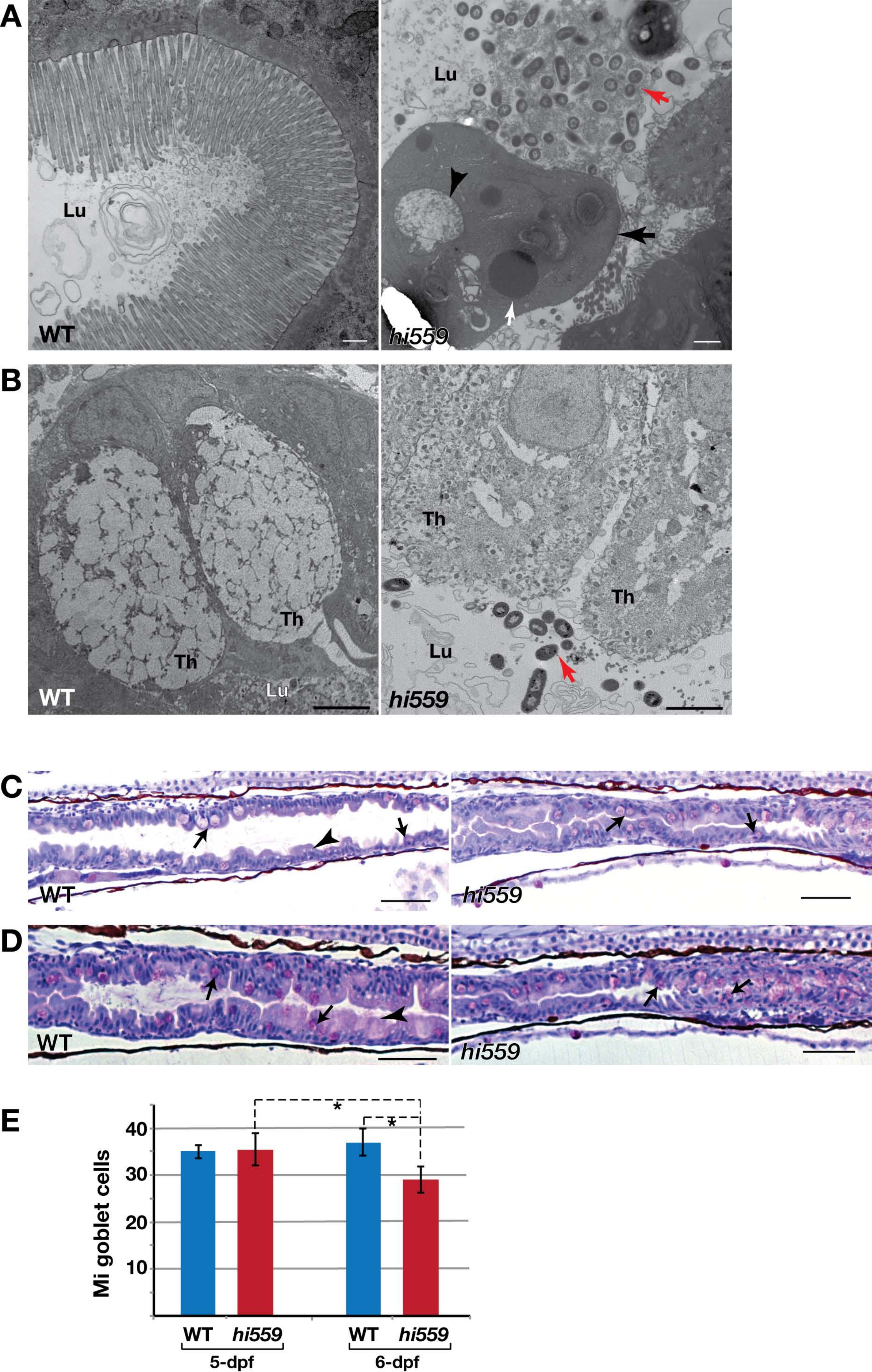

A
Lu
WT
Lu
hi559
B
Th
Th
Lu
WT
Th
Th
Lu
hi559
C
WT
hi559
D
WT
hi559
E
Mi goblet cells
40
30
20
10
*
*
WT
hi559
5-dpf
WT
hi559
6-dpf

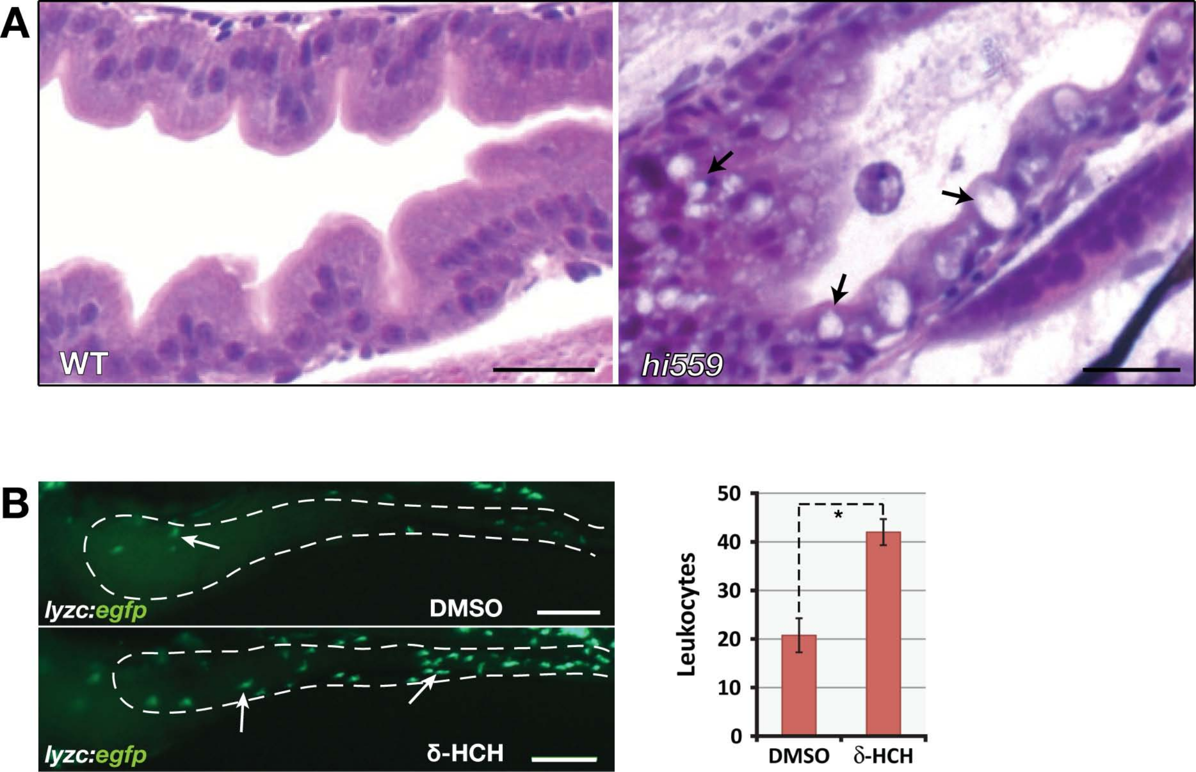
A
WT
hi559
B
lyzc:egfp
DMSO
lyzc:egfp
δ-HCH
Leukocytes
50
40
30
20
10
0
*
DMSO
δ-HCH

A

Symbol	Entrez Gene Name	*P*-value
C4B	complement component 4B (Chido blood group)	3.39E-05
CFB	complement factor B	4.14E-05
SOCS3	suppressor of cytokine signaling 3	6.62E-04
C3	complement component 3	4.88E-04
HPX	hemopexin	4.00E-02
CEBPB	CCAAT/enhancer binding protein (C/EBP), beta	2.37E-03
FGA	fibrinogen alpha chain	2.77E-03
CP	ceruloplasmin (ferroxidase)	3.28E-02
C9	complement component 9	1.33E-03
FGB	fibrinogen beta chain	2.68E-04
FGG	fibrinogen gamma chain	3.45E-04
CRP	C-reactive protein, pentraxin-related	2.32E-02
JUN	jun proto-oncogene	6.24E-04
STAT3	signal transducer and activator of transcription 3	2.47E-03
A2M	alpha-2-macroglobulin	1.53E-02
HMOX2	heme oxygenase (decycling) 2	4.84E-02
PLG	plasminogen	1.15E-02
NRAS	neuroblastoma RAS viral (v-ras) oncogene homolog	2.74E-02
SERPING1	serpin peptidase inhibitor, clade G (C1 inhibitor)	4.01E-02

B

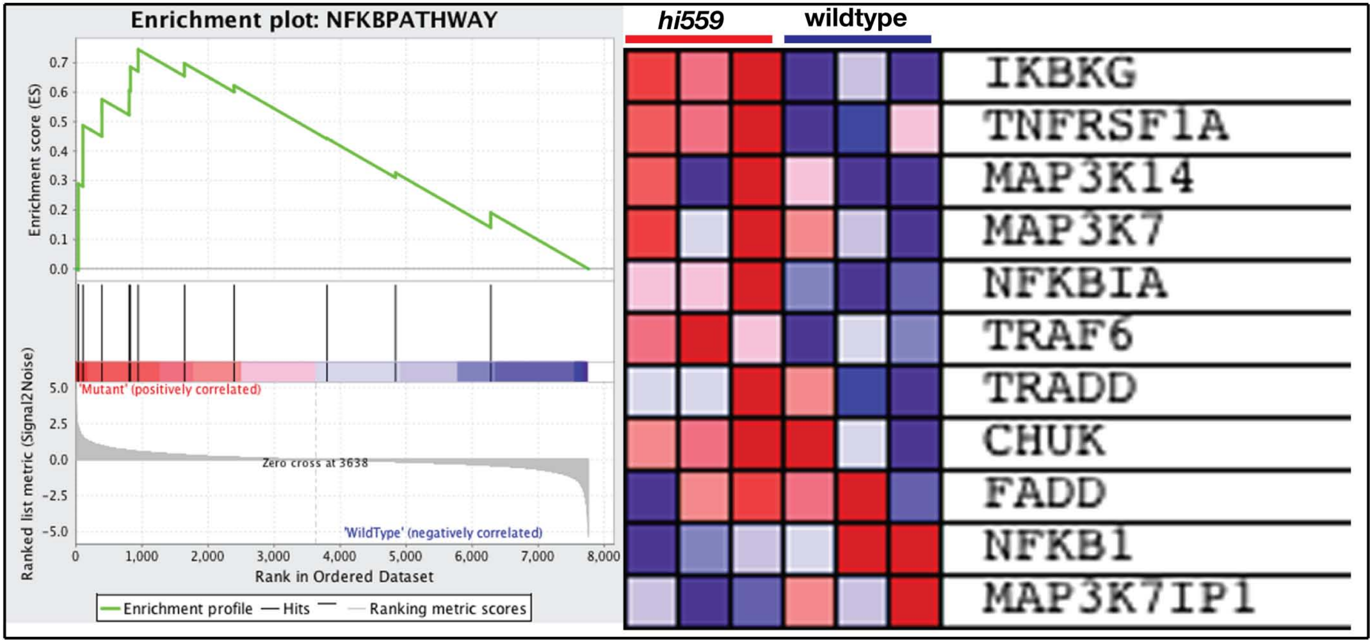

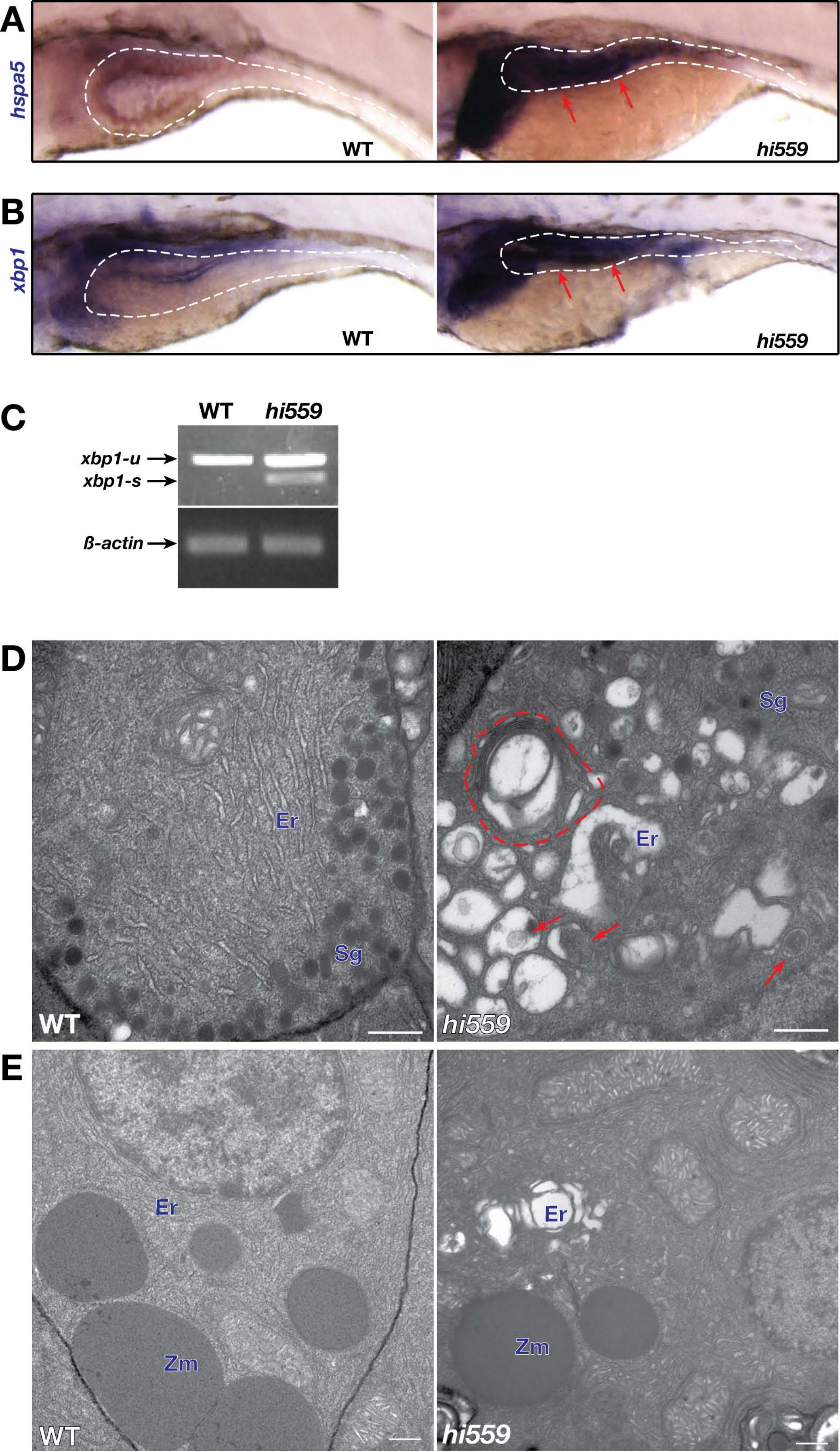

A
hspa5
WT
hi559
B
xbp1
WT
hi559
C
WT
hi559
xbp1-u
xbp1-s
ß-actin
D
Er
Sg
WT
Sg
Er
hi559
E
Er
Zm
WT
Er
Zm
hi559

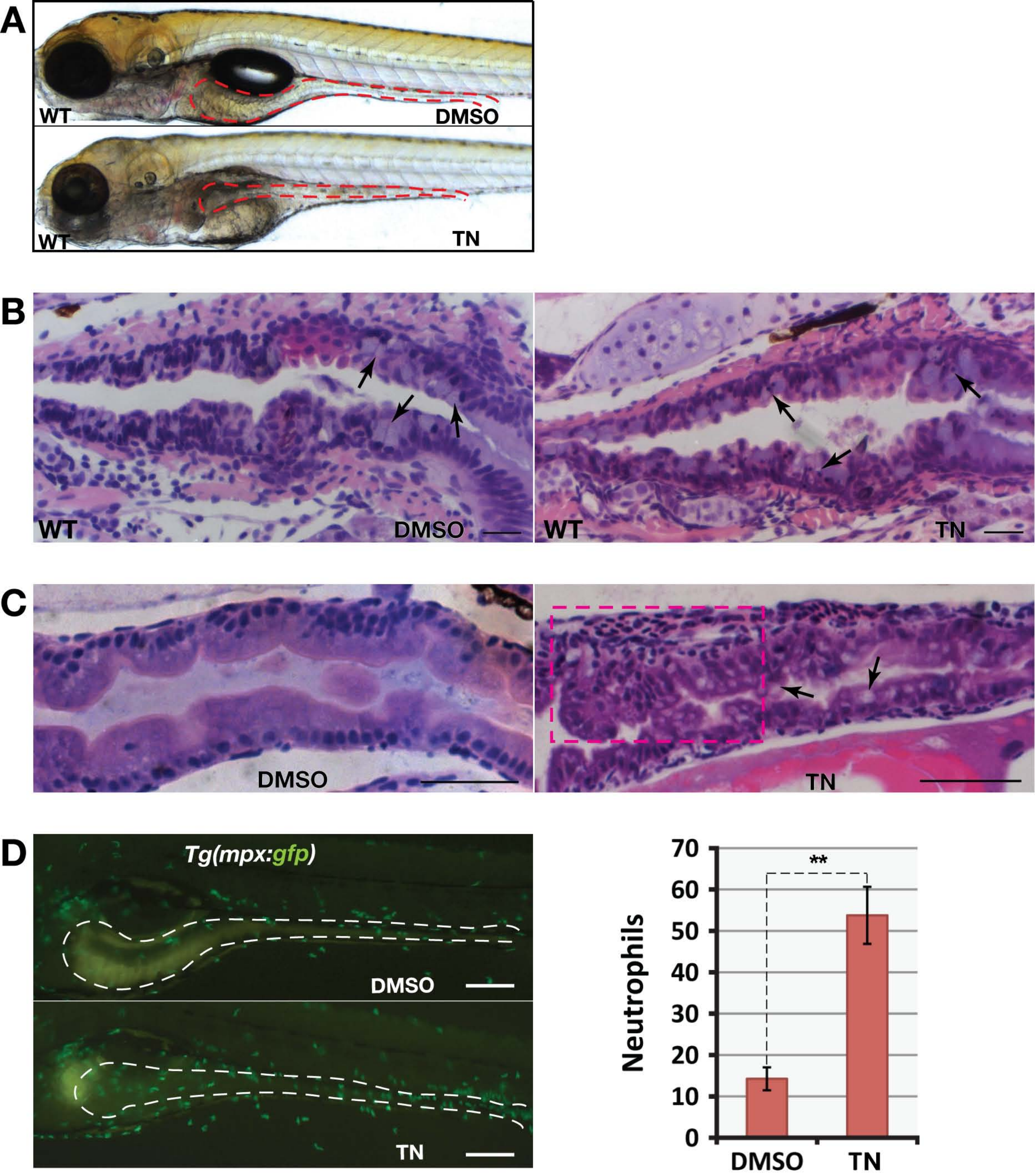

A
WT
DMSO
WT
TN
B
WT
DMSO
WT
TN
C
DMSO
TN
D
Tg(mpx:gfp)
DMSO
TN
Neutrophils
**
0
10
20
30
40
50
60
70
DMSO
TN

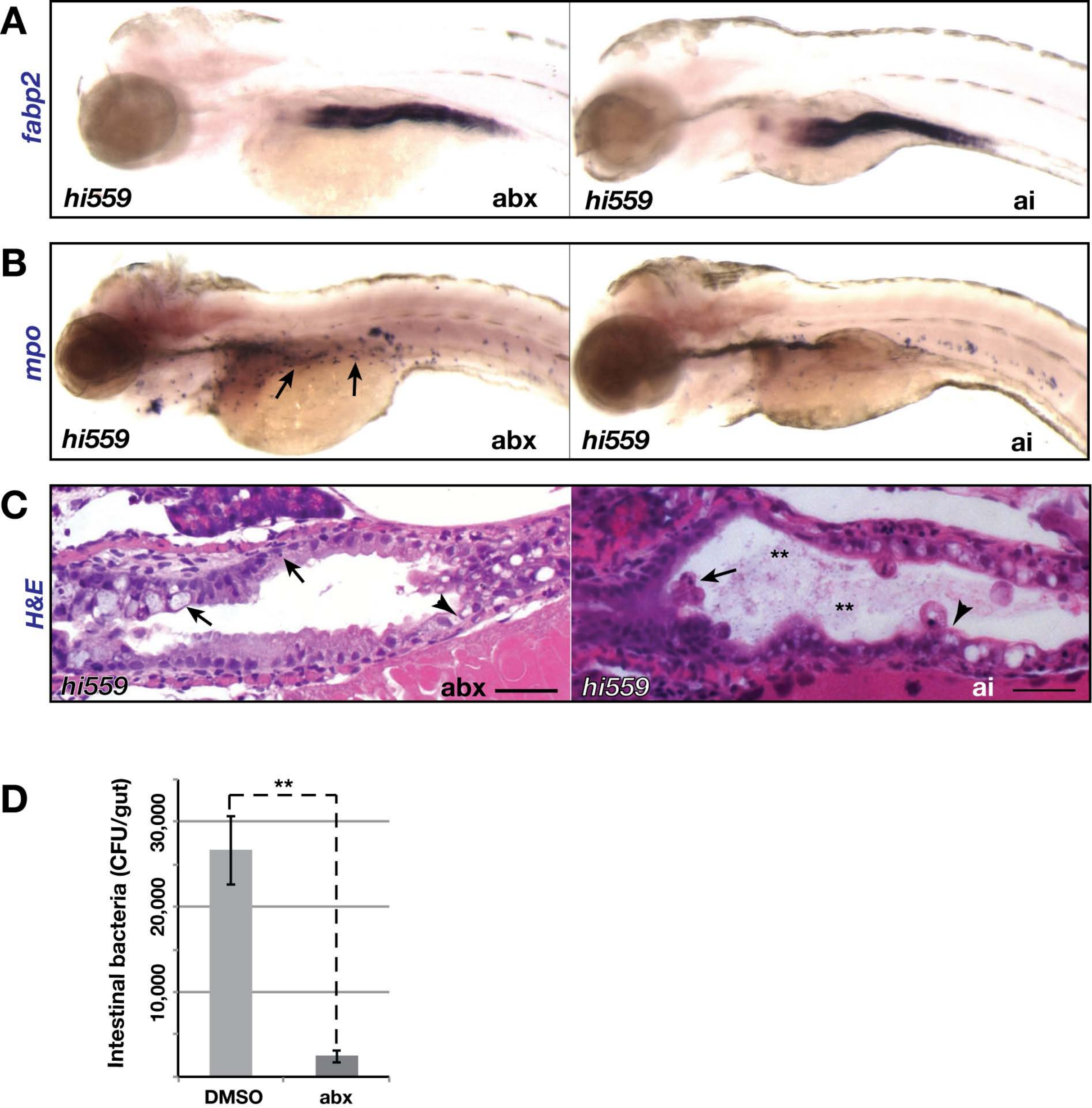
A
fabp2
hi559
abx
hi559
ai
B
mpo
hi559
abx
hi559
ai
C
H&E
**
**
hi559
abx
hi559
ai
D
**
Intestinal bacteria (CFU/gut)
30,000
20,000
10,000
DMSO
abx
